# Engineered Glycosidases for the Synthesis of Analogs of Human Milk Oligosaccharides

**DOI:** 10.3390/ijms23084106

**Published:** 2022-04-07

**Authors:** Pavlína Nekvasilová, Michaela Hovorková, Zuzana Mészáros, Lucie Petrásková, Helena Pelantová, Vladimír Křen, Kristýna Slámová, Pavla Bojarová

**Affiliations:** 1 Laboratory of Biotransformation, Institute of Microbiology of the Czech Academy of Sciences, Vídeňská 1083, CZ-14220 Praha, Czech Republic; pavlina.nekvasilova@biomed.cas.cz (P.N.); michaela.hovorkova@biomed.cas.cz (M.H.); zuzana.meszaros@biomed.cas.cz (Z.M.); petraskova@biomed.cas.cz (L.P.); kren@biomed.cas.cz (V.K.); slamova@biomed.cas.cz (K.S.); 2 Department of Genetics and Microbiology, Faculty of Science, Charles University, Viničná 5, CZ-12843 Praha, Czech Republic; 3 Faculty of Food and Biochemical Technology, University of Chemistry and Technology Prague, Technická 1903/3, CZ-16628 Praha, Czech Republic; 4 Laboratory of Molecular Structure Characterization, Institute of Microbiology of the Czech Academy of Sciences, Vídeňská 1083, CZ-14220 Praha, Czech Republic; pelantova@biomed.cas.cz

**Keywords:** enzymatic synthesis, glycosidase, human milk oligosaccharide, mutagenesis

## Abstract

Enzymatic synthesis is an elegant biocompatible approach to complex compounds such as human milk oligosaccharides (HMOs). These compounds are vital for healthy neonatal development with a positive impact on the immune system. Although HMOs may be prepared by glycosyltransferases, this pathway is often complicated by the high price of sugar nucleotides, stringent substrate specificity, and low enzyme stability. Engineered glycosidases (EC 3.2.1) represent a good synthetic alternative, especially if variations in the substrate structure are desired. Site-directed mutagenesis can improve the synthetic process with higher yields and/or increased reaction selectivity. So far, the synthesis of human milk oligosaccharides by glycosidases has mostly been limited to analytical reactions with mass spectrometry detection. The present work reveals the potential of a library of engineered glycosidases in the preparative synthesis of three tetrasaccharides derived from lacto-*N*-tetraose (Galβ4GlcNAcβ3Galβ4Glc), employing sequential cascade reactions catalyzed by β3-*N*-acetylhexosaminidase BbhI from *Bifidobacterium bifidum*, β4-galactosidase BgaD-B from *Bacillus circulans*, β4-*N*-acetylgalactosaminidase from *Talaromyces flavus*, and β3-galactosynthase BgaC from *B. circulans*. The reaction products were isolated and structurally characterized. This work expands the insight into the multi-step catalysis by glycosidases and shows the path to modified derivatives of complex carbohydrates that cannot be prepared by standard glycosyltransferase methods.

## 1. Introduction

Human milk oligosaccharides (HMOs) represent a large group of unique lactose-based carbohydrate structures contained in human breast milk. They are abundant, occurring at concentrations of 5–20 g/L [1]. HMOs consist of five monosaccharide units, namely β-d-glucose (Glc), β-d-galactose (Gal), *N*-acetyl-β-d-glucosamine (GlcNAc), α-l-fucose (Fuc), and *N*-acetyl-α-d-neuraminic acid (Neu5Ac). All HMOs are structurally based on lactose, which can be extended with β-(1→3)-linked lacto-*N*-biose (type I HMO) or β-(1→3)/β-(1→6)-linked *N*-acetyllactosamine (type II HMO) at the non-reducing end. Lactose and longer HMO chains can be additionally decorated with Fuc and/or Neu5Ac [2].

In vivo, HMOs are synthesized by glycosyltransferases [3], which are usually strictly selective toward their donors and acceptors, and use costly sugar nucleotides as glycosyl donors. In vitro, several strategies for the synthesis of HMOs have been published, e.g., chemical synthesis, synthesis in cell factories, or enzymatic synthesis [4]. Glycosidase-catalyzed synthesis of HMOs is an alternative method to the use of glycosyltransferases. It may bring several advantages, such as donor/acceptor flexibility, cheap and readily available donor substrates, broader enzyme availability, and higher stability. However, on the other hand, yields of glycosidase-catalyzed reactions are generally lower compared to glycosyltransferase-catalyzed reactions due to the transglycosylation/hydrolysis competition [4,5]. Additionally, in contrast to glycosyltransferases, the broad substrate specificity of glycosidases is sometimes accompanied with a lower regioselectivity.

Lacto-*N*-triose II (LNT2, GlcNAcβ3Galβ4Glc) is a backbone precursor of HMOs composed of lactose and β3-linked *N*-acetylglucosamine at the non-reducing end. Its synthesis can be performed in vitro by β-*N*-acetylhexosaminidases (EC 3.2.1.52, GH20), typical *exo*-enzymes, which naturally cleave *N*-acetylglucosamine and *N*-acetylgalactosamine from the non-reducing end of various *N*-acetyl-β-hexosaminides [6], but may be persuaded into the synthetic mode by an apt choice of reaction conditions. A promising β-*N*-acetylhexosaminidase for the synthesis of this bond was previously identified in *Bifidobacterium bifidum* (BbhI; GH20) [7]. The transglycosylation potential of WT BbhI was examined by Chen et al. [7] using *p*NP-β-GlcNAc as a donor and lactose as an acceptor. Schmölzer et al. chose the strategy of site-directed mutagenesis of BbhI [8]. Lacto-*N*-triose II was produced in high yields using mutant D746E BbhI based on *p*NP-β-GlcNAc donor or GlcNAc-oxazoline donor. Later on, Chen et al. subjected the BbhI domain to targeted mutagenesis, developing three multiple mutants with increased transglycosylation abilities, and used them in the synthesis of lacto-*N*-triose in high yields [9]. Teze et al. (2021) used sequence-based protein engineering for identifying conserved amino acid residues, and replaced them with their structural analogs [10]. Six single mutants were developed and tested for lacto-*N*-triose II synthesis. Another promising β3-regioselective β-*N*-acetylhexosaminidase was discovered in *Tyzerella nexilis* (formerly *Clostridium nexile*) [11].

A β4-regioselective β-*N*-acetylhexosaminidase with a high transglycosylation potential and a broad substrate specificity was found in the filamentous fungus *Talaromyces flavus* [12,13,14]. β-*N*-Acetylhexosaminidases with a dual donor specificity are characterized by their GalNAcase/GlcNAcase ratio, which for *Tf*Hex equals 1.2 [15]. We previously found that the specificity of *Tf*Hex in favor of either GlcNAcase or GalNAcase can be efficiently modulated by site-directed mutagenesis targeted at the E546 residue close to the substrate C-4 hydroxy group. The E546Q variant of *Tf*Hex with a 5.5-fold higher selectivity toward GalNAc was prepared and used for the synthesis of complex hexosamines [16].

Further elongation of lacto-*N*-triose II affording lacto-*N*-tetraose [17] or lacto-*N*-neotetraose [11,17] requires synthetically efficient β3 or β4-galactosidases. The most promising enzyme for this aim is β-galactosidase from *Bacillus circulans* (BgaD, EC 3.2.1.23, GH2). BgaD post-translationally forms four isoforms, namely isoform A (BgaD-A, 189 kDa), isoform B (BgaD-B, 155 kDa), isoform C (BgaD-C, 132 kDa), and isoform D (BgaD-D, 92 kDa) [18,19,20], which largely differ in their transglycosylation activities [20]. The BgaD-D isoform was used in the synthesis of lacto-*N*-neotetraose with a lactose donor and lacto-*N*-triose acceptor by Liu et al. [11] and Zeuner et al. [21]. Notably, the lacto-*N*-neotetraose yields reported by these groups were only obtained from high-performance liquid chromatography (HPLC) analysis, not by isolating the product. Moreover, BgaD-D is well known for its lower regioselectivity, often forming complex product mixtures [22].

In the present work, we demonstrate the preparative synthesis of two basic HMO tetrasaccharides, lacto-*N*-tetraose and lacto-*N*-neotetraose, using β3-selective β-*N*-acetylhexosaminidase from *B. bifidum* and engineered β-galactosidases from *B. circulans* (BgaC [23] and BgaD-B [24]). Furthermore, we focused on the challenging preparation of a lacto-*N*-neotetraose analog, complex GalNAcβ4GlcNAcβ3Galβ4Glc tetrasaccharide, using engineered E546Q variant of β-*N*-acetylhexosaminidase from *T. flavus* with increased selectivity toward GalNAc substrates [16]. All oligosaccharide products were isolated and characterized by nuclear magnetic resonance (NMR), high-resolution mass spectrometry (HRMS), and HPLC. Besides their use as food additives, they are valuable tools in biomedical research. HMOs and their tetrasaccharide analogs are a part of many vital biological structures like glycosphingolipids [25] and important recognition elements of the immune system. Alone or attached to multivalent carriers or in the form of glyco-arrays, they may act as efficient ligands of human lectins with a therapeutic and/or diagnostic potential [26].

## 2. Results

### 2.1. β3-N-Acetylhexosaminidase BbhI and the Synthesis of Lacto-N-triose II (***3***)

**Scheme 1:**
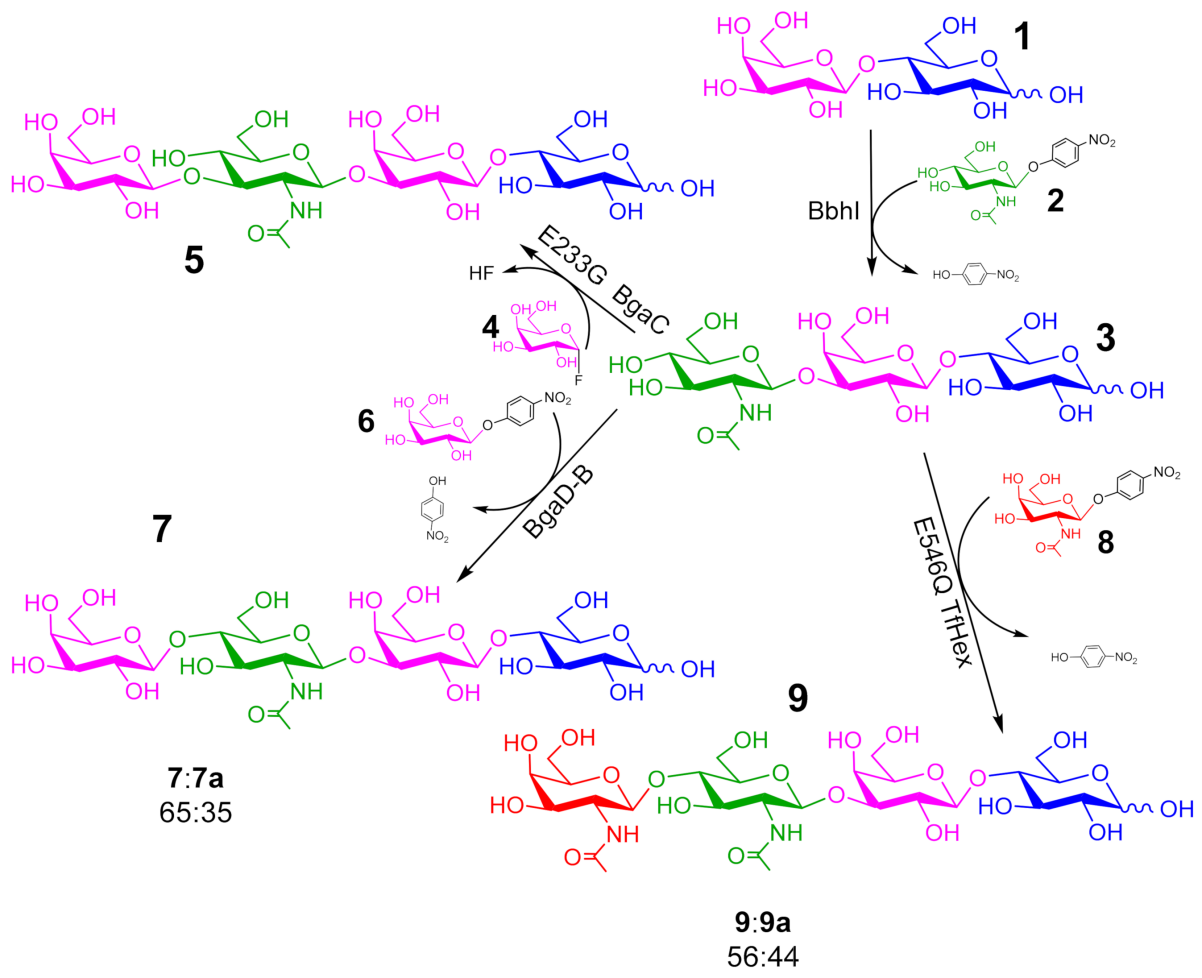
Synthesis of analogs of human milk oligosaccharides by selected glycosidase variants. The color code is as follows: Glc in blue, Gal in magenta, GlcNAc in green, and GalNAc in red. Side products were formed in reactions catalyzed by BgaD-B (**7a**, Galβ6GlcNAcβ3Galβ4Glc) and E546Q *Tf*Hex (**9a**, GlcNAcβ4GlcNAcβ3Galβ4Glc) in the ratios, as indicated in the Scheme.

Lacto-*N*-triose II (GlcNAcβ3Galβ4Glc, **3**) was a key intermediate in the enzymatic synthesis of the target tetrasaccharides—lacto-*N*-tetraose (**5**), lacto-*N*-neotetraose (**7**), and its derivative **9** (Scheme 1). This trisaccharide can be obtained in a transglycosylation reaction using a *p*NP-β-GlcNAc donor and lactose acceptor under the catalysis by a β3-specific β-*N*-acetylhexosaminidase BbhI from *B. bifidum* [7,8]. At first, we expressed BbhI in *E. coli* as a C-terminal His_6_-tagged protein. However, unfortunately, purification by metal affinity chromatography never afforded an enzyme preparation void of the contaminating β-galactosidase activity (ca 10% related to BbhI activity), originating apparently from the host LacZ β-galactosidase. This was a major drawback for the transglycosylation reactions, as the acceptor disaccharide lactose was cleaved by this contaminating enzyme. Therefore, we decided to change the expression host for the methylotrophic yeast *Pichia pastoris*, in which we have already successfully expressed a number of glycosidases [27,28]. In this expression system, the enzyme was extracellularly produced and purified, affording 3.5 mg of protein from 1 L cultivation media, with a specific activity of 18.8 U/mg GlcNAcase and 25.7 U/mg GalNAcase. The transglycosylation reaction for the synthesis of lacto-*N*-triose II (**3**) catalyzed by BbhI was optimized under monitoring by HILIC-HPLC (20 mM *p*NP-β-GlcNAc and 400 mM lactose), and the typical isolated yield of trisaccharide **3** reached ca. 50% after its purification by gel filtration. The integrity and purity of compound **3** were confirmed by HPLC, NMR, and HRMS (see Supplementary Materials).

### 2.2. Galactosynthase E233G BgaC and the Synthesis of Lacto-N-tetraose (***5***)

For the selective introduction of β-(1→3)-galactopyranosyl moiety to lacto-*N*-triose II (**3**) yielding lacto-*N*-tetraose (**5**, Galβ3GlcNAcβ3Galβ4Glc) we used the previously described regioselective E233G variant of β3-galactosynthase from *B. circulans* (BgaC) [23], the construct of which was developed by Li and Kim [29]. Glycosynthases catalyze the glycosyl transfer using an activated substrate of the opposite anomeric configuration, such as α-galactosyl fluoride in the case of β-galactosynthases, in a single step without hydrolyzing the product. In the case of BgaC, the substitution of the catalytic-nucleophile glutamate at position 233 with glycine, typical for glycosynthases, resulted in the loss of the enzyme hydrolytic activity and in its ability to process α-d-galactosyl fluoride for quantitative β3-galactosylations. The expression of BgaC in *E. coli* and purification by affinity chromatography were performed as described previously [30]. The yield of the production was 9.4 g cells from 1 L of medium and 48.7 mg of protein from 1 g cells. We verified the rescue of hydrolytic activity of E233G BgaC, as described previously [31,32]. In the transgalactosylation reaction, α-Gal-F (**4**) [31] was used as a glycosyl donor and lacto-*N*-triose II (**3**) as an acceptor in a molar ratio of 1:1. The reaction ran until the fluoride donor was fully consumed (ca. 50 h). The tetrasaccharide lacto-*N*-tetraose (**5**) was obtained as a white powder in a high yield (21 mg, 71% isolated yield) and with 100% regioselectivity. The integrity and purity of compound **5** were confirmed by HPLC, NMR, and HRMS (see the Supplementary Materials).

### 2.3. β-Galactosidase BgaD-B and the Synthesis of Lacto-N-neotetraose (***7***)

β-Galactosidase from *B. circulans* isoform B (BgaD-B) was selected for the synthesis of tetrasaccharide **7** due to its reported high regioselectivity for β4-glycosidic bonds and good transglycosylation potential [24]. *B. circulans* produces four isoforms of β-galactosidases (BgaD-A, -B, -C, and -D), which originate by posttranslational cleaving of the C-terminus of isoform A by an endogenous protease [19,20]. We prepared the truncated isoform BgaD-B [20] by deletion of the longer isoform BgaD-A [31] via site-directed mutagenesis using 5′-phosphorylated primers for PCR, followed by ligation of the linearized cropped gene. The plasmid carrying the cropped gene (pCold II-DNA-bgaD-B) was used for producing the enzyme in *E. coli* BL21 Gold (DE3). The correct folding of this large protein was ensured by a low expression temperature (15 °C), enabled by the promoter of cold-shock protein A (*cspA*). Moreover, the low temperature impeded the co-expression of the intrinsic LacZ β-galactosidase, as confirmed by us previously [31]. The enzyme purification was accomplished by Ni^2+^ affinity chromatography. The β-galactosidase BgaD-B was stable after dialysis in pH 6 for several months at 4 °C. The typical yield of the production was 8 g of cells from 1 L media, affording 14.8 mg of protein from 1 g of cells, with a specific hydrolytic activity of 3.2 U/mg (for *p*NP-β-Gal, **6**). The resulting BgaD-B sample was confirmed by SDS-PAGE to be void of contaminating LacZ.

The synthesis of lacto-*N*-neotetraose (**7**, Galβ4GlcNAcβ3Galβ4Glc) was accomplished from lacto-*N*-triose II (**3**) as a glycosyl acceptor under the catalysis by the prepared BgaD-B, using *p*NP-β-Gal (**6**) as a glycosyl donor. In the optimization analytical reactions, BgaD-B showed a better performance and a higher glycosylation yield than its longer isoform BgaD-A. The isolated yield of **7** from the preparative reaction was 28.7 mg (23%). Full structural characterization of product **7** was performed (see the Supplementary Materials). Regrettably, NMR analysis revealed that the regioselectivity of BgaD-B was not as exclusive as expected, and as also observed in the galactosylations of monosaccharide acceptors. The isolated tetrasaccharide **7** contained contamination by its β-1,6-isomer **7a** (see the Supplementary Materials). Neither a lengthy optimization of the reaction (changing the donor/acceptor ratio, testing various co-solvent systems) improved the regioselectivity of the enzyme, and the side product Galβ6GlcNAcβ3Galβ4Glc (**7a**) could not be separated either on gel chromatography or by preparative HPLC. Thus, we conclude that BgaD-B was not able to catalyze the β4-galactosylation of lacto-*N*-triose II (**3**) with exclusive regioselectivity.

### 2.4. β4-N-Acetylgalactosaminidase E546Q TfHex and the Synthesis of Tetrasaccharide (***9***)

For the synthesis of novel tetrasaccharide **9**, we opted for our previously described mutant variant E546Q β-*N*-acetylhexosaminidase from *T. flavus* [16]. The point mutation ensured that the original promiscuous enzyme *Tf*Hex WT (GalNAcase/GlcNAcase activity ratio 1.2 [16]) became a quite selective GalNAcase, with this ratio shifted to 7 (14% of the GlcNAcase activity maintained). The preparative production of E546Q *Tf*Hex mutant variant in *P. pastoris* was essentially performed as described previously [16]. The use of a minimal cultivation medium ensured that the extracellularly produced enzyme could be easily purified to homogeneity in a single purification step by cation-exchange chromatography (pH 3.5). The purification afforded 52 mg of protein from 200 mL cultivation media with 0.5 U/mg GlcNAcase activity and 3.5 U/mg GalNAcase activity. This mutant variant was used to introduce the GalNAc terminal subunit at the non-reducing end of lacto-*N*-triose II (**3**) for the production of lacto-*N*-neotetraose analog **9** (GalNAcβ4GlcNAcβ3Galβ4Glc). In the GalNAcylation reaction, *p*NP-β-GalNAc (**8**) was used as a glycosyl donor. The reaction catalyzed by E546Q *Tf*Hex afforded the desired tetrasaccharide **9** in a total isolated yield of 27% (6 mg). However, the NMR analysis showed that the product contained the contamination of the GlcNAc-terminated tetrasacharide (GlcNAcβ4GlcNAcβ3Galβ4Glc, **9a**). This side product could not be detected by either MS or HPLC methods, despite extensive optimizations of the latter. Apparently, despite the relatively high selectivity toward the GalNAc moiety of the E546Q variant, and despite the exclusive β4-regioselectivity of *Tf*Hex in transglycosylation reactions [33], in this particular reaction, the enzyme cleaved the β3-bound GlcNAc moiety of lacto-*N*-triose II (**3**) and formed GlcNAc-terminated tetrasaccharide **9a** (see also the Supplementary Materials, Scheme S1). This behavior is quite unique and points to the phylogenetic origin of β-*N*-acetylhexosaminidases as chitinolytic enzymes. It appears that the selective production of the GalNAcβ4GlcNAcβ3Galβ4Glc motif (**9**) is an extremely challenging task that might even be a bottleneck in the synthesis of this type of structure by glycosidases. Moreover, the selective GalNAcase activity is extremely rare in β-*N*-acetylhexosaminisases possessing transglycosylation capabilities [4], and the possibilities of protein engineering may have their limits in this respect.

## 3. Discussion

HMOs are a desired commodity in glycobiology, and, therefore, novel methods for their synthesis, preferably large-scale, high-yielding, and selective, are in high demand. Unlike established glycosyltransferases, glycosidases offer priceless toleration of structural variations in both donor and acceptor molecules, which may be further exploited in biomedical research. It should be noted that many previous publications dealing with the synthesis of HMOs by glycosidases rely exclusively on the interpretation of ESI-MS and/or of HPLC data [34,35]. This can severely distort the impression of the true catalytic potential of the enzymes in this field, because, as exemplified in this work, the possible formation of closely structurally related regioisomers may not be reliably detectable by either of these methods. Hence, complex NMR analysis of the isolated products is probably the only “gold-standard” method to provide real answers on the usefulness of the respective synthetic approaches, as also shown by Vuillemain et al. [36].

The present work highlights the need to produce synthetic enzymes of a high purity and selectivity. The problem of the co-expression and co-purification of intrinsic LacZ β-galactosidase in enzyme samples has emerged in our previous work [31] and needs to be carefully addressed in order to avoid substrate depletion or undesirable side reactions. We solved this problem either by using an alternative expression host of *P. pastoris* or by using the pCOLD vector for the production in *E. coli* at a low temperature. Both methods offer an additional benefit of better protein folding in the case of large or complex proteins.

BbhI, even as WT, proves to be an efficient tool for the glycosylation of Gal-terminated acceptors with a high regioselectivity. Interestingly, this property is not shared in the chitinolytic enzymes of the GH20 β-*N*-acetylhexosaminidase family, which hardly accept galactose as a glycosyl acceptor [37], contrary to other representatives of this family [38].

An excellent performance in terms of 100% regioselectivity and quantitative yield was demonstrated in the previously underestimated β3-galactosynthase BgaC that afforded lacto-*N*-tetraose (**5**). Thus, this synthetic approach to lacto-*N*-tetraose is highly competitive compared with other glycosidase-catalyzed routes to this product described previously [34,35]. Advantageously, the α-Gal-F donor employed in this synthesis is readily available in multigram amounts using our optimized procedure [31]. The commercially available *p*-nitrophenyl glycoside donors used in all other enzyme syntheses are ca 5–20-fold cheaper than the respective sugar nucleotides.

The lack of regioselectivity of β4-galactosylation in the glycosidase family is one bottleneck in the synthesis of HMOs. Additionally, the longer isoforms of *B. circulans* β-galactosidases prospective for selective disaccharide formation cannot be transformed into galactosynthases due to their particular structural relations in their active site [31], which further limits their applicability. Due to the low regioselectivity of hydrolysis by glycosidases, the cleavage of the product mixture results in high losses. This problem is even more pronounced when aiming at selective GalNAc-ylation. A satisfactory solution to this problem will require further intensive studies in the future.

## 4. Materials and Methods

### 4.1. Standard Assays for Enzyme Activity, Protein Concentration and Purity

The hydrolytic activity of E546Q *Tf*Hex [16], BgaD-B [31], and BbhI [39] was determined by an end-point assay with spectrophotometric detection. The respective *p*-nitrophenyl substrates, namely *p*NP-β-GlcNAc (**2**), *p*NP-β-Gal (**6**), and *p*NP-β-GalNAc (**8**), were used. The reaction mixture (50 μL) composed of 2 mM *p*NP-substrate and buffer, i.e., citrate-phosphate buffer (50 mM citric acid/100 mM Na_2_HPO_4_, pH 5) for *Tf*Hex, citrate-phosphate buffer (50 mM citric acid/100 mM Na_2_HPO_4_, pH 6) for BbhI, and sodium phosphate buffer (50 mM NaH_2_PO_4_/50 mM Na_2_HPO_4_, pH 6) for BgaD-B, was incubated at 35 °C in a thermoshaker and the reaction was started by adding 10 μL of an appropriately diluted enzyme, so that the reaction conversion did not exceed 10%. The activity assay ran for 10 min at 850 rpm and was stopped by adding 1 mL of 0.1 M sodium carbonate. The liberated *p*-nitrophenol in the form of *p*-nitrophenolate formed under basic conditions was then detected spectrophotometrically at 420 nm. In acidophilic glycosidases, continuous activity assays are less sensitive at a narrow range of substrate concentrations due to the interference of the substrate absorbance, and hence we opted for a discontinuous assay.

The catalytic activity of the synthase E233G BgaC was determined based on the rescue of hydrolytic activity in the presence of a small external nucleophile [31]. An exchange of the glutamate residue for a small non-nucleophilic residue (G or A) in the active site of the enzyme creates a cavity, into which a small external nucleophile—sodium azide or sodium formate—can enter and enable the processing of the hydrolytic substrate [32]. The rescue of the hydrolytic activity was accomplished by adding the exogenous nucleophile (sodium formate) in a concentration of 0.5–7.5 M to the standard enzyme activity assay, as described above, using 2 mM *p*NP-β-Gal (**6**) substrate in a sodium phosphate buffer (50 mM NaH_2_PO_4_/50 mM Na_2_HPO_4_, pH 6.5).

The concentration of enzymes was determined by the Bradford method [40] using Protein Assay Dye Reagent Concentrate (Bio-Rad, Watford, UK) according to bovine plasma γ-globulin (IgG, Bio-Rad, Watford, UK) calibration. The purity of the enzymes was verified by SDS-PAGE.

### 4.2. Preparation, Expression, and Purification of BbhI

The gene encoding for β-*N*-acetylhexosaminidase from *B. bifidum* JCM 1254 (BbhI; GenBank ID: AB504521 [7]) was prepared synthetically on a commercial basis (Generay Biotech, Shanghai, China), cloned into the yeast expression vector pPICZαA (*Eco*RI/*Kpn*I) and extracellularly expressed in *P. pastoris* KM71H (Invitrogen, Waltham, MA, USA) as previously described for fungal β-*N*-acetylhexosaminidases [28]. Briefly, the pPICZαA-BbhI was electroporated (Electroporator Bio-Rad, Hercules, CA, USA) into the competent cells *P. pastoris* KM71H according to the manufacturer’s instructions (EasySelect *Pichia* Expression Kit, Invitrogen, Waltham, MA, USA), and the electroporated cells were cultivated in various concentrations under the selection pressure of zeocin (100 μg/mL, Invitrogen, Waltham, MA, USA) on YPD agar plates for 3 days at 28 °C. To screen the production of BbhI in *P. pastoris* KM71H on a small scale, a combination of BMGY (Buffered Glycerol Complex Medium) and BMMY (Buffered Methanol Complex Medium) was used and the expression of BbhI was induced by methanol (0.5 % *v*/*v*) every 24 h. On day 5 after inoculation, the cultures were tested for the presence of BbhI by 8% SDS-PAGE and enzyme activity assay using the *p*NP-β-GlcNAc substrate. Clones with the highest hexosaminidase activity were cryopreserved at −80 °C and employed in the large-scale production of the enzyme. For the preparatory production of BbhI, BMGH (1 L; Buffered Glycerol Minimal Medium) and BMMH (200 mL; Buffered Methanol Minimal medium) were used, starting with a 5 h preculture in YPD medium (15 mL, 10 g/L yeast extract, 2 g/L bacteriological peptone, 2 g/L d-glucose). After three days of induction, BbhI was purified from the culture media by cation exchange chromatography (Fractogel EMD-SO^3−^, Merck, Darmstadt, Germany) employing the Äkta Purifier chromatography system (GE Healthcare, Chicago, IL, USA), as described previously [28]. The protein concentration was determined according to Bradford and the purity of protein fractions was determined by SDS-PAGE using 8% polyacrylamide gel. Fractions comprising BbhI were pooled and concentrated; the buffer was exchanged for 50 mM sodium citrate-phosphate pH 6, and the purified enzyme was stored at 4 °C for several weeks.

### 4.3. Preparation, Expression, and Purification of BgaD-B

The cropped isoform of β-galactosidase from *B. circulans* BgaD-B [20] was prepared by deletion of the C-terminal domain of the longer isoform BgaD-A [31]. The N-terminal His-tagged construct of the β-galactosidase from *B. circulans* BgaD-A (pCold II-bgaD-A) cloned in the expression vector pCold II DNA using the restriction sites *Sac*I/*Eco*RI was prepared commercially (Generay Biotech, Shanghai, China). The isolated plasmid pCold II-bgaD-A was used as a template in the PCR reaction to obtain the shorter isoform, BgaD-B. The deletion of BgaD-A was performed by site-directed mutagenesis using the pair of 5′-phosphorylated PCR primers (Generi Biotech, Hradec Králové, Czech Republic): Fw: 5′-TAAGAATTCAAGCTTGTCGACCTGCAGTCTAGA-3′, Re: 5′-CATTCTAATATCCTCGACCGCAACAGCCC-3′ and Phusion Site-Directed Mutagenesis Kit (ThermoFisher Scientific, Waltham, MA, USA). The reaction mixture (50 µL) contained 1 ng of pCold II-bgaD-A template, 0.5 µM of each primer (0.5 µL), and dNTP mixture (1 µL; 200 µM each) provided within the kit. The polymerase chain reaction was performed using high-fidelity Pfu-based DNA polymerase (Phusion Hot Start DNA Polymerase; 0.5 μL; 2 U/μL) from the kit in a T-Personal Thermal Cycler (Biometra, Göttingen, Germany) with the following steps: initial denaturation at 98 °C for 30 s, followed by 30 cycles of denaturation at 98 °C for 10 s, primer annealing at 68 °C for 20 s, elongation at 72 °C for 5 min, and a final extension at 72 °C for 5 min. Then, the linear chains of the PCR product were ligated to the circular plasmid. The ligation mixture (7.5 µL) containing 20 ng of the PCR product (3 µL) and T4 DNA ligase provided by the kit (0.5 µL) was centrifuged and incubated for 10 min at room temperature. It was then used for the transformation of *E. coli* Top10 competent cells. The transformation was accomplished by heat shock at 42 °C for 75 s. After 1 h regeneration at 37 °C, the cells were spread on an agar plate with ampicillin (100 μg/mL, Serva, Heidelberg, Germany) as a selection factor. Plasmids were isolated from the transformed *E. coli* colonies with the High Pure Plasmid Isolation Mini Kit (Roche, Basilea, Swiss). The plasmid containing the desired cropped gene was amplified and purified with the Genopure Plasmid Midi Kit (Roche, Basilea, Swiss). The isolated plasmid was fully sequenced to confirm the presence of the bgaD-B gene and the integrity of the plasmid.

For the production of the BgaD-B variant, the *E. coli* BL21 Gold (DE3) cells (Takara Bio., Kusacu, Japan) were transformed with pCold II-bgaD-B. The production and purification were performed essentially as described previously [31]. Transformed *E. coli* cells were inoculated into 20 mL of Luria–Bertani medium (LB, 10 g/L tryptone, 5 g/L yeast extract, and 5 g/L NaCl) containing 100 μg/mL ampicillin in 100 mL flasks, and incubated at 37 °C, 120 rpm overnight. Then, the precultures were transferred to 200 mL of LB medium with 100 μg/mL ampicillin in 1 L flasks and incubated at 37 °C at 200 rpm. After 3–4 h, the optical density of the growing culture at 600 nm (OD_600_) increased from 0.3 to 0.9, and isopropyl 1-thio-β-d-galactopyranoside (IPTG, 0.1 mM) was added to induce the expression of the enzyme. Then, the flasks were incubated at 15 °C at 200 rpm. After 24 to 26 h, the cells were harvested by centrifugation at 8880× *g* for 20 min at 4 °C, and were frozen and stored at −20 °C.

The purification of BgaD-B was performed by Ni^2+^ affinity chromatography using the Äkta Prime Plus purification system (GE Healthcare, Miami, FL, USA). Equilibration buffer (30 mL, 20 mM NaH_2_PO_4_/20 mM Na_2_HPO_4_, pH 7.4) and phenylmethylsulfonyl fluoride (PMSF, 1 mM) were added to the harvested cells. The cell suspension was sonicated using UltraSonic Processor UP50 H (Ultrasound Technologies, Caldicot, UK) for six cycles (each of 1 min with 2 min break on ice). Then, the suspension was centrifuged at 20,230× *g* for 20 min at 4 °C. The cell-free extract was loaded on an equilibrated HisTrap^TM^ column (GE Healthcare, Miami, FL, USA) and washed with an equilibration buffer. The enzyme elution was performed by washing the column with an elution buffer containing 100 mM imidazole and 500 mM NaCl. Fractions were collected and analyzed for protein content using the Bradford assay, the purified enzyme was pooled, dialyzed against 2 × 5 L of 50 mM sodium phosphate buffer pH 6, and stored at 4 °C.

### 4.4. Expression and Purification of E233G BgaC

The gene of a β3-galactosynthase E233G BgaC cloned in the vector pET-Duet^TM^-1 (*Bam*HI/*Pst*I) [23] was kindly donated by Prof. Elling, RWTH Aachen, DE. The production and purification were performed according to Kamerke et al. [30]. The pET-Duet-1-E233G-bgaC plasmid was used for the transformation of *E. coli* BL21 Gold (DE3) cells (Takara Bio., Shanghai, China). Transformed cells were inoculated into LB medium (60 mL) containing 100 μg/mL ampicillin in 0.5 L flasks, and were incubated at 37 °C and 220 rpm overnight. This pre-culture was used for the inoculation of the TB medium (600 mL; 24 g/L yeast extract, 12 g/L tryptone, 4 mL/L glycerol, 17 mM KH_2_PO_4_, and 72 mM K_2_HPO_4_ pH 7.5) containing 100 μg/mL ampicillin in 3 L flasks, and was incubated at 37 °C and 150 rpm until OD_600_ reached 0.6–0.8. Then, the enzyme expression was induced by 0.5 mM IPTG and the cultures were grown for 24 h at 25 °C and 150 rpm. The cells were harvested by centrifugation at 8880× *g* for 20 min at 4 °C, and were frozen and stored at −20 °C. For purification, the cells were suspended in the equilibration buffer (20 mM phosphate/500 mM NaCl, 20 mM imidazole, pH 7.4) with PMSF (1 mM), and were sonicated (1 min pulse, 2 min pause, six repetitions). After centrifugation (20,230× *g*, 20 min, and 4 °C), the supernatant was loaded on an equilibrated HisTrap™ column. The enzyme was eluted with an elution buffer (20 mM phosphate/500 mM NaCl/500 mM imidazole, pH 7.4). Fractions were collected and analyzed for protein content using the Bradford assay; the purified E233G BgaC (68.5 kDa) was pooled, dialyzed against 2 × 5 L of 50 mM sodium phosphate buffer pH 6.5, and stored at 4 °C.

### 4.5. Expression and Purification of E546Q TfHex

The gene of the E546Q *Tf*Hex variant cloned in the pPICZαa expression vector (*Eco*RI/*Kpn*I) was prepared commercially (Generay Biotech, Shanghai, China), and was electroporated into *P. pastoris* KM71H competent cells using the EasySelect *Pichia* Expression Kit, as described previously [16]. For preparatory production, cryopreserved cultures (100 μL) were inoculated into 15 mL of YPD medium, this pre-culture was incubated at 28 °C at 220 rpm for 5 h. Then, the pre-culture was inoculated into a BMGH medium and incubated under the same conditions overnight. On day 2, the culture was centrifuged (5000 rpm, 10 min, and 12 °C) and the pellet was resuspended in the BMMH medium and was incubated for three more days under the induction of methanol (0.5 % *v*/*v*) every 24 h.

On day 5, the grown culture was centrifuged (5000 rpm, 10 min, 12 °C) and the supernatant was purified by cation exchange chromatography (Fractogel EMD SO^3−^, Merck, Germany) using an Äkta Purifier system (GE Healthcare, Miami, FL, USA). The column equilibration was performed with 10 mM sodium citrate-phosphate buffer pH 3.5. The enzyme was eluted by a linear gradient of 0–1 M NaCl (60 mL), concentrated by ultrafiltration in 50 mM sodium citrate-phosphate buffer, and stored at 4 °C.

### 4.6. Synthesis of Oligosaccharides

#### 4.6.1. 2-Acetamido-2-deoxy-β-d-glucopyranosyl-(1→3)-β-d-galactopyranosyl-(1→4)-d-glucopyranose (**3**, lacto-*N*-triose II, GlcNAcβ3Galβ4Glc)

For optimizing the synthesis of **3** on an analytical scale, the reaction mixtures contained *p*NP-β-GlcNAc (10–40 mM) as a donor, and lactose (100–400 mM) as an acceptor, in 50 mM citrate-phosphate buffer pH 6, and 0.4–1.4 U/mL of enzyme BbhI; the total reaction volume was 0.2 mL. The reactions were incubated at 35 °C with shaking (1000 rpm, Thermomixer Comfort, Eppendorf, Hamburg, Germany). The reaction progress was monitored by HPLC. The optimized reaction conditions were applied for the preparatory synthesis of trisaccharide **3**.

For the preparative reaction, the reaction mixture contained *p*NP-β-GlcNAc (20 mM, 27.4 mg) as a donor and lactose (400 mM, 540 mg) as an acceptor in 50 mM citrate-phosphate buffer pH 6, and 84 µg of BbhI (total reaction volume 4 mL). The reaction was incubated at 35 °C with shaking (1000 rpm) and the progress of the reactions was monitored by HPLC. After 6 h, the reaction was stopped by boiling (99 °C, 5 min) and the products were separated using gel permeation chromatography with Toyopearl HW-65 (Merck, Germany) as a stationary phase and water as a mobile phase (6 mL/min). The fractions were analyzed by TLC. Trisaccharide **3** (22.2 mg, 41 μmol, 51% isolated yield) was lyophilized as a white solid and its structure was confirmed by HRMS and NMR (see the Supplementary Materials).

#### 4.6.2. β-d-Galactopyranosyl-(1→3)-2-acetamido-2-deoxy-β-d-glucopyranosyl-(1→3)-β-d-galactopyranosyl-(1→4)-d-glucopyranose (**5**, lacto-*N*-tetraose, Galβ3GlcNAcβ3Galβ4Glc)

The title compound **5** was synthesized after optimization on an analytical scale. The reaction mixtures contained α-Gal-F donor (**4**; 10 mM; prepared as described previously [31]), lacto-*N*-triose II acceptor (**3**; 10 mM in 50 mM sodium phosphate buffer pH 6.5), and β3-galactosynthase E233G BgaC (4.5–9 mg/mL). The reactions were incubated at 35 °C with shaking (850 rpm) and were monitored by TLC. The optimized reaction conditions were applied for the preparatory synthesis of tetrasaccharide **5**.

For the preparative reaction, the glycosyl donor α-Gal-F (**4**; 10 mM, 7.47 mg) and the acceptor lacto-*N*-triose II (**3**; 10 mM, 22.7 mg) were dissolved in 50 mM sodium phosphate buffer pH 6.5, and β3-galactosynthase E233G BgaC (36 mg, 525 µM, 1.8 mL) was added (total reaction volume of 4.2 mL) at 35 °C and 850 rpm. The reaction was monitored by TLC. As donor **4** is degraded under these conditions after a couple of hours, after 6 h, another portion of **4** (3.6 mg) was added. When the donor was consumed (ca after 48 h), the reaction was stopped by enzyme denaturation at 99 °C for 2 min and centrifuged at 12,100× *g* for removing the denatured enzyme. The supernatant was purified by gel permeation chromatography (Biogel P2) at a flow rate of 5.5 mL/h with water as a mobile phase. The fractions containing the pure product were pooled and lyophilized. The title compound **5** was obtained as a white fluffy solid. The isolated yield was 21 mg (29.7 mmol, 71% isolated yield). For the structural analysis (HRMS, NMR), see the Supplementary Materials.

#### 4.6.3. β-d-Galactopyranosyl-(1→4)-2-acetamido-2-deoxy-β-d-glucopyranosyl-(1→3)-β-d-galactopyranosyl-(1→4)-d-glucopyranose (**7**, lacto-*N*-neotetraose, Galβ4GlcNAcβ3Galβ4Glc)

The title compound **7** was synthesized after optimization on an analytical scale. The analytical reactions were composed of a *p*NP-β-Gal donor (**6**; 15–50 mM) and lacto-*N*-triose II acceptor (**3**; 50–100 mM) in 50 mM sodium phosphate buffer pH 6, and β4-galactosidase BgaD-B (5–100 mU/mL). In parallel, the analytical reactions were performed with the longer isoform BgaD-A prepared as described previously [31], and compared. The reactions were incubated at 35 °C with shaking (1000 rpm) and monitored by TLC and HPLC. The optimized reaction conditions were applied for the preparatory synthesis of tetrasaccharide **7**. To suppress the formation of by-product **7a**, a range of donor/acceptor ratios (1:1–6.7) and enzyme concentrations (5–100 mU/mL) were tested, as well as acetonitrile co-solvent (20–40% *v*/*v*).

For the preparative reaction, the glycosyl donor *p*NP-β-Gal (**6**; 30 mM, 27 mg) and the acceptor lacto-*N*-triose II (**3**; 100 mM, 162 mg) were dissolved in 50 mM sodium phosphate buffer pH 6, and β4-galactosidase BgaD-B (94.5 µg, 75 mU, 15 µL) was added (total reaction volume 3 mL) at 35 °C and 1000 rpm. The reaction was monitored by TLC. After 1.5 h, another portion of donor **6** (27 mg) was added. When over 90% of the donor was consumed (ca. after 6 h), the reaction was stopped by enzyme denaturation at 99 °C for 2 min and centrifuged at 12,100× *g* for removing the denatured enzyme. The supernatant was purified by gel permeation chromatography (Biogel P2, Bio-Rad, Hercules, CA, USA) at a flow rate of 7.2 mL/h with water as a mobile phase. The fractions containing the product were pooled and lyophilized. The title compound **7** was obtained as a white fluffy solid. The isolated yield was 28.7 mg (40.6 mmol, 23% isolated yield). For the structural analysis (HRMS, NMR), see Supplementary Materials. It was found that by-product **7a** was formed in this reaction (**7**/**7a** = 65/35).

#### 4.6.4. 2-Acetamido-2-deoxy-β-d-galactopyranosyl-(1→4)-2-acetamido-2-deoxy-β-d-glucopyranosyl-(1→3)-β-d-galactopyranosyl-(1→4)-d-glucopyranose (**9**, GalNAcβ4GlcNAcβ3Galβ4Glc)

The title compound **9** was synthesized after optimization on an analytical scale. The reaction mixtures contained *p*NP-β-GalNAc donor (**8**; 30 mM) and lacto-*N*-triose II acceptor (**3**; 100–500 mM in a mixture of acetonitrile and 50 mM citrate-phosphate buffer pH 5), and E546Q *Tf*Hex (1–5 U/mL). The reactions were incubated at 35 °C with shaking (1000 rpm) and monitored by TLC and HPLC. The optimized reaction conditions were applied for the preparatory synthesis of tetrasaccharide **9**.

For the preparative reaction, *p*NP-β-GalNAc donor (**8**, 10 mg, 29 μmol), lacto-*N*-triose II acceptor (**3**; 54 mg, 72 μmol), and E546Q *Tf*Hex (20 μL, 2.5 U, 0.8 mg) were incubated in 30 % acetonitrile in 50 mM citrate-phosphate buffer pH 5 (total reaction volume 1 mL) at 35 °C and 1000 rpm. The progress of the reaction was monitored by TLC (propane-2-ol/water/NH_4_OH aq., 7/2/1). The reaction was stopped after 5.5 h by boiling for 2 min and centrifuged (12,100× *g*, 10 min) for removing the denatured protein. The supernatant was separated by gel permeation chromatography (Biogel P-2) with water as the mobile phase at an elution rate of 7.1 mL/h. The title compound **9** was obtained as a white solid (6 mg, 0.8 μmol, 27% isolated yield). For the structural analysis (HRMS and NMR), see Supplementary Materials. It was found that by-product **9a** was formed in this reaction (**9**/**9a** = 56/44).

## 5. Conclusions

In this work, we demonstrate the potential of a tailored library of mutant and native glycosidases (EC 3.2.1.)—originally hydrolases—to replace the commonly used glycosyltransferases in the complex task of synthesizing HMOs and their analogs. The concerted action of four enzymes, β3-*N*-acetylhexosaminidase from *B. bifidum*, β4-galactosidase BgaD-B from *B. circulans*, β4-*N*-acetylgalactosaminidase from *T. flavus*, and β3-galactosynthase BgaC from *B. circulans,* resulted in the preparation of lacto-*N*-triose II, lacto-*N*-tetraose, and lacto-*N*-neotetraose, and the yet undescribed GalNAc-terminated lacto-*N*-neotetraose analog. To improve the reaction specificity, regioselectivity, and yield, and to suppress the side hydrolysis, point mutations were introduced to the native glycosidase variants. Additionally, the selectivity and yield of enzyme production were optimized by careful selection of the expression hosts and cloning approach. All oligosaccharides were isolated and purified on a preparative scale for the complete structural characterization. At the same time, the present methodology points to the bottlenecks of the glycosidase approach, especially concerning the lack of selectivity in the case of lacto-*N*-neotetraose, and its GalNAc-capped analog, which may deserve further research in the future. On the other hand, the production of lacto-*N*-triose II and lacto-*N*-tetraose may be considered as a competitive alternative to the glycosyltransferase approach, with the additional advantage of glycosidases to tolerate possible functionalizations in the substrate molecule as abundantly exemplified earlier.

## Data Availability

Data are available from the authors on request.

## Supplementary Materials

The following supporting information can be downloaded at: https://www.mdpi.com/article/10.3390/ijms23084106/s1.

## Abbreviations

BbhI	*Bifidobacterium bifidum* β-*N*-acetylhexosaminidase
BgaC	*Bacillus circulans* β3-galactosidase
BgaD	*Bacillus circulans* β4-galactosidase
ESI	electrospray ionization
Fuc	l-fucose
Gal	d-galactose
GH	glycoside hydrolase
Glc	d-glucose
GlcNAc	*N*-acetyl-d-glucosamine
GlcNAc-oxazoline	1,2-dideoxy-2′-methyl-α-d-glucopyranoso-[2,1-*d*]-Δ2′-oxazoline
HILIC	hydrophilic interaction liquid chromatography
HPLC	high performance liquid chromatography
HMO	human milk oligosaccharide
HRMS	high resolution mass spectrometry
IPTG	isopropyl 1-thio-β- d-galactopyranoside
LNnT	lacto-*N*-neotetraose (β-d-Gal-(1→4)-β-d-GlcNAc-(1→3)-β-d-Gal-(1→4)-d-Glc)
LNT	lacto-*N*-tetraose (β-d-Gal-(1→3)-β-d-GlcNAc-(1→3)-β-d-Gal-(1→4)-d-Glc)
LNT2	lacto-*N*-triose II (β-d-GlcNAc-(1→3)-β-d-Gal-(1→4)-d-Glc)
MS	mass spectrometry
Neu5Ac	*N*-acetyl-α-d-neuraminic acid
NMR	nuclear magnetic resonance
PCR	polymerase chain reaction
*p*NP-Gal	4-nitrophenyl β-d-galactopyranoside
*p*NP-GlcNAc	4-nitrophenyl 2-acetamido-2-deoxy-β- d-glucopyranoside
