# Supplementary material for: Engineered Glycosidases for the Synthesis of Analogs of Human Milk Oligosaccharides
Source: Int J Mol Sci. 2022 Apr 7;23(8):4106. doi: 10.3390/ijms23084106 (PMC9027921; doi:10.3390/ijms23084106)
Supplement: Supplementary file 1 [file ijms-23-04106-s001.zip › ijms-1655410-supplementary.pdf]

International Journal of
Molecular Sciences

MDPI

Supplementary Materials

Engineered Glycosidases for the Synthesis of Analogs of Human Milk Oligosaccharides

Pavlína Nekvasilová [1, 2], Michaela Hovorková [1,2], Zuzana Mészáros [1, 3], Lucie Petrásková [1], Helena Pelantová [4], Vladimír Křen [1], Kristýna Slámová [1], and Pavla Bojarová [1,*]

[1] Laboratory of Biotransformation, Institute of Microbiology of the Czech Academy of Sciences, Vídeňská 1083, CZ-14220 Praha 4, Czech Republic; pavlina.nekvasilova@biomed.cas.cz (P.N.); michaela.hovorkova@biomed.cas.cz (M.H.); zuzana.meszaros@biomed.cas.cz (Z.M.); petraskova@biomed.cas.cz (L.P.); slamova@biomed.cas.cz (K.S.); kren@biomed.cas.cz (V.K.)

[2] Department of Genetics and Microbiology, Faculty of Science, Charles University, Viničná 5, CZ-12843, Praha 2, Czech Republic

[3] Faculty of Food and Biochemical Technology, University of Chemistry and Technology Prague, Technická 1903/3, CZ-16628 Praha 6, Czech Republic

[4] Laboratory of Molecular Structure Characterization, Institute of Microbiology of the Czech Academy of Sciences, Vídeňská 1083, CZ-14220 Praha 4, Czech Republic; pelantova@biomed.cas.cz (H.P)

* Correspondence: bojarova@biomed.cas.cz (P.B.); Tel.: +420-296-442-360

Abstract: Enzymatic synthesis is an elegant biocompatible approach to complex compounds such as human milk oligosaccharides (HMOs). These compounds are vital for healthy neonatal development with a positive impact on the immune system. Though HMOs may be prepared by glycosyltransferases, this pathway is often complicated by the high price of sugar nucleotides, stringent substrate specificity, and low enzyme stability. Engineered glycosidases (EC 3.2.1) represent a good synthetic alternative especially if variations in the substrate structure are desired. Site-directed mutagenesis can improve the synthetic process with higher yields and/ or increased reaction selectivity. So far, the synthesis of human milk oligosaccharides by glycosidases was limited to analytical reactions with mass spectrometry detection. The present work reveals the potential of a library of engineered glycosidases in the preparative synthesis of three tetrasaccharides derived from lacto-*N*-tetraose (Galβ4GlcNAcβ3Galβ4Glc), employing sequential cascade reactions catalyzed by β3-*N*-acetylhexosaminidase BbhI from *Bifidobacterium bifidum*, β4-galactosidase BgaD-B from *Bacillus circulans*, β4-*N*acetylgalactosaminidase from *Talaromyces flavus*, and β3-galactosynthase BgaC from *B. circulans*. The reaction products were isolated and structurally characterized. This work expands the insight into the multi-step catalysis by glycosidases and shows the path to modified derivatives of complex carbohydrates that cannot be prepared by standard glycosyltransferase methods.

Content:

1. Materials and Methods

2. Characterization of Enzyme Variants

3. Structural Characterization of Prepared Compounds

4. References

1. Materials and Methods

General

Thin-layer chromatography (TLC) was performed on Merck silica gel DC-Alufolien Kieselgel 60 F254 using a mobile phase of isopropanol/water/ammonium, 7:2:1. Enzyme substrates *p*NP-Gal, *p*NP-GalNAc and *p*NP-GlcNAc were from Gold Biotechnology, USA. Glycosynthase substrate α-Gal-F was synthesized according to Hovorková et al. [1]. If not stated otherwise, all other chemicals were from VWR Chemicals or Lach-Ner (Czech Republic).

HPLC Analysis

The Shimadzu Prominence LC analytical system comprised Shimadzu CBM-20A system controller, Shimadzu LC-20AD binary HPLC pump, Shimadzu CTO-10AS column oven, Shimadzu SIL-20ACHT cooling autosampler, and Shimadzu SPD-20MA diode array detector (Shimadzu, Kyoto, JP). Analyses were performed on a TSK gel Amide-80 column (250 4.6 mm, 5 m) preceded by TSKgel Amide-80 Guardgel (3.2 15 mm, Tosoh corp., JP) with gradient elution as follows (A = acetonitrile, B = water): 22 % B for 0-7 min, 22-35 % B for 7-20 min, 35 % B for 20-25 min, 35-22 % B for 25-26 min, and 22 % B for next 9 min for column equilibration; at a flow rate of 1 mL/min at 25 °C; injection volume 1 µL; samples were dissolved in acetonitrile/ water (4/1, *v*/*v*). Detection was performed at 200 nm.

HRMS Analysis

Mass spectra were measured using LTQ Orbitrap XL hybrid mass spectrometer (Thermo Fisher Scientific, USA), ionization was performed with an electrospray ion source. Methanol/water (4:1, v/v) was used as a mobile phase at a flow rate of 100 µL/min. The samples were dissolved in methanol or methanol/water and injected using a 5-µL loop into the mobile phase flow. Mass spectra were measured in the positive-ion mode under the following conditions: spray voltage 5.0 kV, capillary voltage 9 V, tube lens voltage 150 V, and capillary temperature 275 °C. The spectra were recorded at a resolution of 100,000.

NMR Analysis

NMR data were acquired on a Bruker Avance III 400 MHz (compound **3**), and 700 MHz (compounds **5**, **7**, **9**, and **10**) spectrometer (Bruker BioSpin, Rheinstetten, Germany) in D_2O (99.96 atom % D, VWR Chemicals, Leuven, Belgium) at 30 °C. The proton spectra were referenced using the residual signal of D_2O (δ_H 4.732 ppm); the carbon spectra to the signal of acetone (δ_C 30.50 ppm). Due to the huge overlap of proton signals, the individual monosaccharide units were assigned using COSY, HSQC, 1d-TOCSY, and HSQC-TOCSY experiments. The position of *N*-acetyls was proved by the upfield shift of attached carbons C-2 and by HMBC contacts between carbonyls and corresponding methines H-2. The type of glycosidic linkage (1→3, 1→4 or 1→6) was confirmed using the HMBC correlation of involved carbons C-3, C4 or C-6 with the anomeric proton of adjacent monosaccharide.

2. Characterization of Enzyme Variants

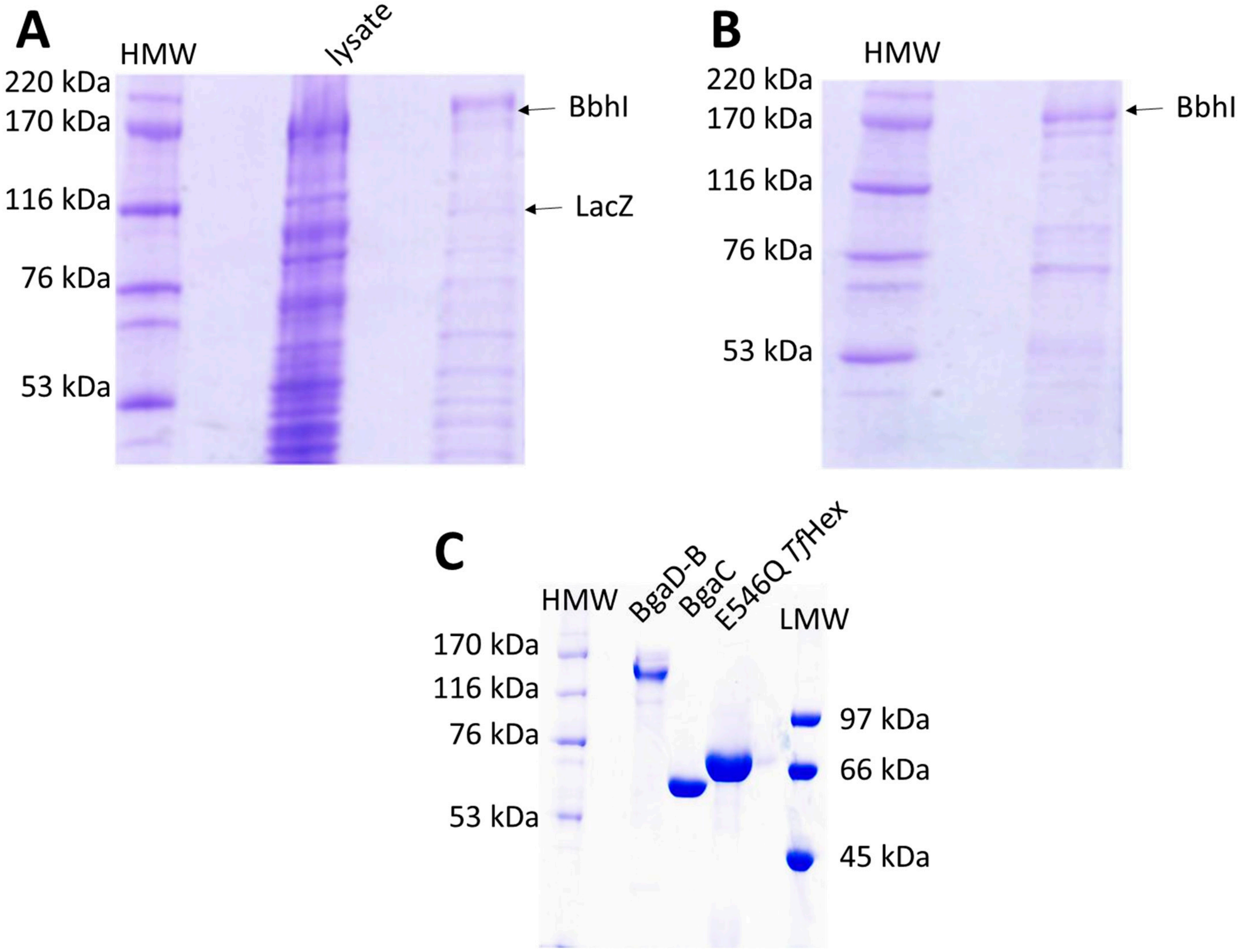

Figure S1. SDS-PAGE of the prepared enzymes. (**A**) BbhI (176 kDa) expressed in *E. coli* and purified by HisTrap, (**B**) BbhI expressed in *P. pastoris* and purified by cation-exchange chromatography, (**C**) BgaD-B (155 kDa; the slight impurity has estimated MW of *ca*. 100 kDa and is not LacZ), BgaC (68.5 kDa), and E546Q *Tf*Hex (66 kDa). HMW – High molecular weight marker: 170 kDa – α2-macroglobulin; 116 kDa – β-galactosidase; 76 kDa – transferrin; 53 kDa – glutamate dehydrogenase. LMW – Low molecular weight marker: phosphorylase b from rabbit muscle (97 kDa), bovine serum albumin (66 kDa), chicken egg white ovalbumin (45 kDa).

Production of BbhI in *E. coli*

The gene of β-*N*-acetylhexosaminidase from *B. bifidum* JCM 1254 (BbhI; GenBank ID: AB504521) was prepared commercially (Generay Biotech, Shanghai, CN) and cloned into pET21-b(+) vector. The enzyme was expressed in *E. coli* BL21 pLysS strain under the induction by 0.5 mM IPTG (isopropyl 1-thio-β-D-galactoside, Merck, DE) and purified on a 5 mL HisTrap column (GE Healthcare, US) connected to the Äkta Purifier protein chromatography system (GE Healthcare, US) as described for OGA in our recent work [27]. The fractions containing BbhI in elution buffer were pooled, 5× diluted with 100 mM Tris/HCl buffer pH 7.4 containing 100 mM NaCl and concentrated and re-buffered using Amicon Ultra Centrifugal Filters (Merck, DE) to remove excess imidazole from elution buffer. The purified enzyme was stored at 4 °C for several months without any significant loss of activity.

3. Structural Characterization of Prepared Compounds

Table S1. ^{1}H and ^{13}C NMR data for 2-acetamido-2-deoxy-β-D-glucopyranosyl-(1→3)-β-D-galactopyranosyl-(1→4)-Dglucopyranose (**3**; 399.87 MHz for ^{1}H, 100.55 MHz for ^{13}C, D_2O, 30 °C). ***α*-anomer**

	**Atom**	C	**m.**	H	**n$_H$**	**m.**	***J* [Hz]**	**HMBC[x]**
**Glc[A]**	**1**	92.08	D	5.224	1	d	3.8	
	**2**	71.41	D	3.579	1	dd	9.9, 3.8	
	**3**	71.67	D	3.834	1	dd	9.9, 8.8	
	**4**	78.72	D	3.641	1	dd	10.1, 8.8	1_B
	**5**	70.39	D	3.951	1	ddd	-	1_A
	**6**	60.26	T	3.87[H]	2	m	-	
**Gal[B]**	**1**	103.16	D	4.444	1	d	7.9	
	**2**	70.31	D	3.602	1	dd	9.9, 7.9	
	**3**	82.19	D	3.730	1	dd	9.9, 3.1	1_C
	**4**	68.63	D	4.151	1	br d	3.3	
	**5**	75.16	D	3.72[H]	1	m	-	1_B
	**6**	61.22	T	3.77[H]	2	m	-	

**GlcNAc^{C}**	**1**	103.07	D	4.697	1	d	8.4	
	**2**	55.95	D	3.757	1	dd	10.2, 8.4	
	**3**	73.85	D	3.573	1	dd	10.2, 8.9	
	**4**	69.99	D	3.475	1	m	-	
	**5**	75.94	D	3.45^{H}	1	m	-	1$_{C}$
	**6**	60.79	T	3.901	1	dd	12.4, 2.1	
				3.76^{H}	1	m	-	
	**2-CO**	175.22	S	-	0	-	-	2^{C}, Ac
	**Ac**	22.44	Q	2.042	3	s	-	

β-anomer

	**Atom**	$_{C}$	**m.**	$_{H}$	**n$_{H}$**	**m.**	***J* [Hz]**	**HMBC^{x}**
**Glc^{A}**	**1**	96.01	D	4.665	1	d	8.0	
	**2**	74.07	D	3.284	1	m	-	
	**3**	74.63	D	3.648	1	m	-	
	**4**	78.62	D	3.64^{H}	1	m	-	1^{B}
	**5**	75.06	D	3.61^{H}	1	m	-	1$_{A}$
	**6**	60.39	T	3.955	1	dd	12.3, 2.0	
				3.79^{H}	1	dd	-	
**Gal^{B}**	**1**	103.20	D	4.444	1	d	7.9	
	**2**	70.28	D	3.594	1	dd	9.9, 7.9	
	**3**	82.22	D	3.726	1	dd	9.9, 3.2	1^{C}
	**4**	68.61	D	4.151	1	br d	3.3	

	**5**	75.16	D	3.72^{H}	1	m	-	1$_{B}$
	**6**	61.22	T	3.77^{H}	2	m	-	
**GlcNAc^{C}**	**1**	103.07	D	4.693	1	d	8.4	
	**2**	55.95	D	3.757	1	dd	10.2, 8.4	
	**3**	73.85	D	3.573	1	dd	10.2, 8.9	
	**4**	69.99	D	3.475	1	m	-	
	**5**	75.94	D	3.45^{H}	1	m	-	1$_{C}$
	**6**	60.79	T	3.901	1	dd	12.4, 2.1	
				3.76^{H}	1	m	-	
	**2-CO**	175.22	S	-	0	-	-	2^{C}, Ac
	**Ac**	22.44	Q	2.042	3	s	-	

^{H} HSQC readout; ^{x} diagnostic ^{13}C to ^{1}H HMBC correlations

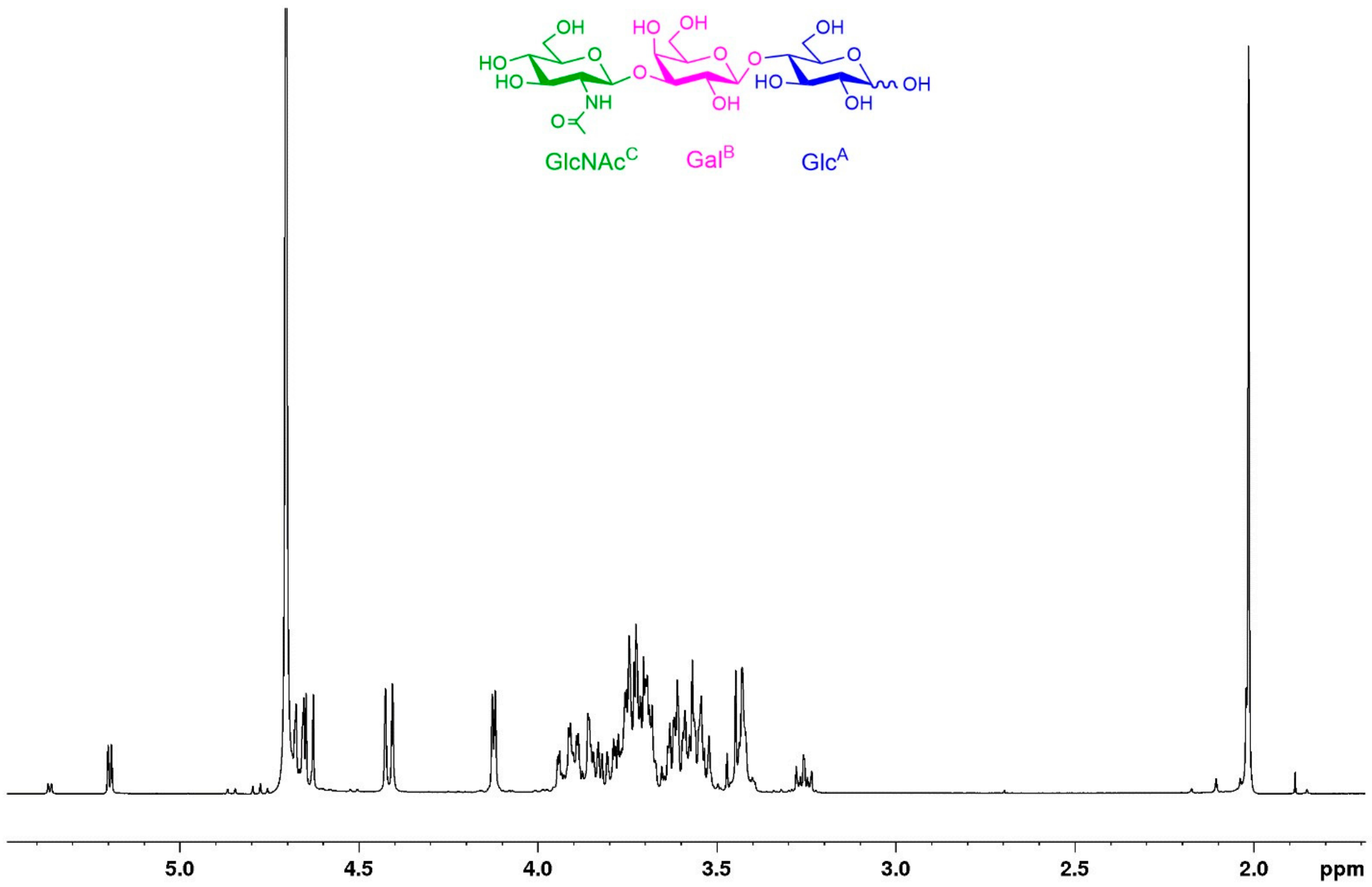

Figure S2a. ^{1}H NMR spectrum of compound **3** (399.87 MHz for ^{1}H, D_2O, 30 °C).

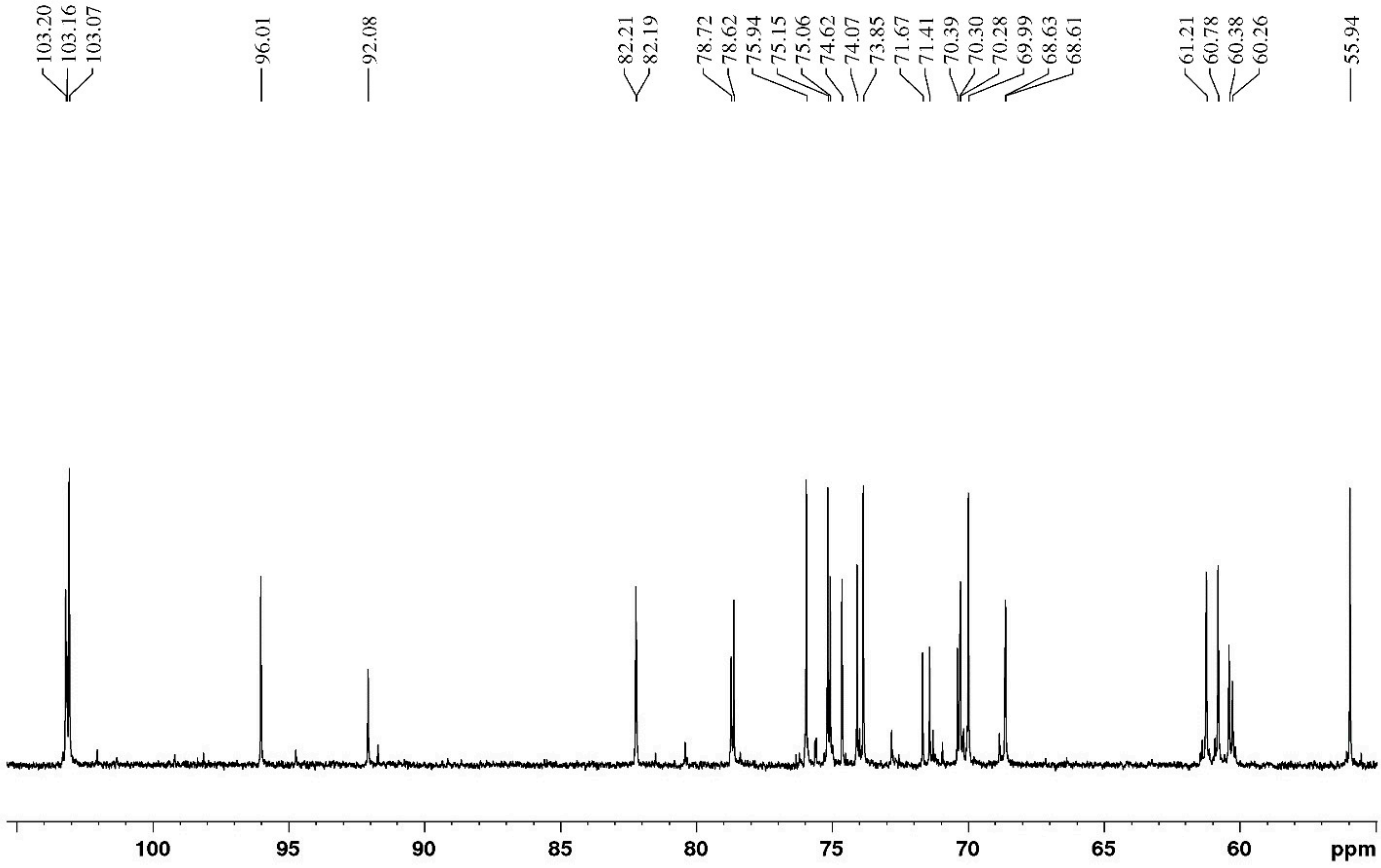

Figure S2b. ^{13}C NMR spectrum of compound **3** – without signals of *N*-acetyls (100.55 MHz for ^{13}C, D_2O, 30 °C).

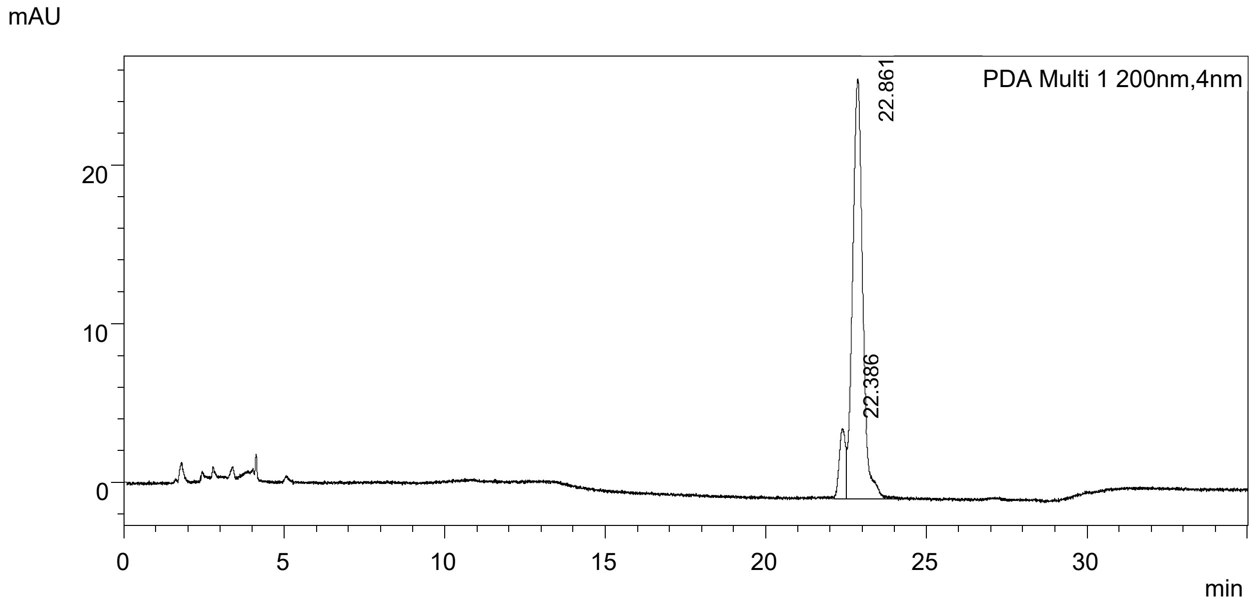

Figure S2c. HPLC chromatogram of isolated compound **3** (RT= 22.861 min, 91% purity).

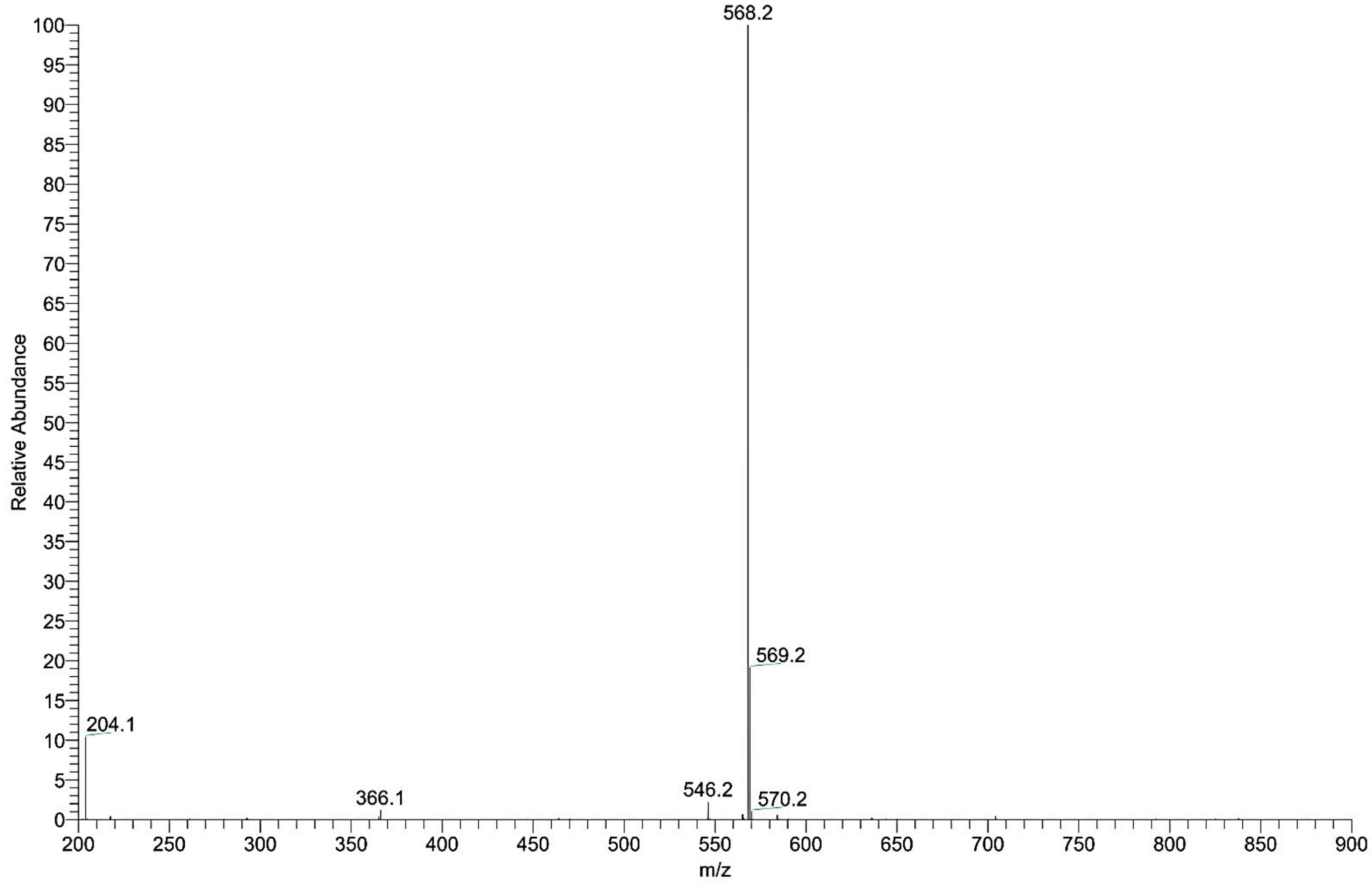

Figure S2d. MS spectrum (ESI+) of compound **3**: [M + Na]+, *m/z* 568.2. HRMS (ESI+): *m/z* for $C_{20}H_{35}O_{16}NNa^+$ calculated 568.18465, found 568.18456 (-0.44 ppm).

Table S2. ^{1}H and ^{13}C NMR data for β-D-galactopyranosyl-(1→3)-2-acetamido-2-deoxy-β-D-glucopyranosyl-(1→3)-β-Dgalactopyranosyl-(1→4)-D-glucopyranose (**5**; 700.13 MHz for ^{1}H, 176.05 MHz for ^{13}C, D_2O, 30 °C).

***α*-anomer**

	Atom	C	m.	H	n_H	m.	*J* [Hz]	HMBC^x
**Glc^A**	**1**	92.01	D	5.221	1	d	3.8	
	**2**	71.34	D	3.578	1	dd	9.9, 3.8	
	**3**	71.60	D	3.830	1	dd	9.9, 8.9	
	**4**	78.65	D	3.641	1	dd	10.1, 8.9	1_B
	**5**	70.32	D	3.948	1	ddd	-	1_A
	**6**	60.18	T	3.88^H	1	m	-	

				3.84^{H}	1	m	-	
**Gal^{B}**	**1**	103.08	D	4.445	1	d	7.9	
	**2**	70.24	D	3.602	1	dd	10.0, 7.9	
	**3**	82.16	D	3.739	1	dd	10.0, 3.2	1$_{C}$
	**4**	68.54	D	4.151	1	br d	3.3	
	**5**	75.09	D	3.71^{H}	1	m	-	1$_{B}$
	**6**	61.14	T	3.78^{H}	1	m	-	
				3.74^{H}	1	m	-	
**GlcNAc^{C}**	**1**	102.72	D	4.742	1	d	8.4	
	**2**	54.90	D	3.896	1	dd	10.3, 8.4	
	**3**	82.31	D	3.822	1	dd	10.3, 8.5	1$_{D}$
	**4**	68.68	D	3.571	1	dd	9.9, 8.5	
	**5**	75.40	D	3.483	1	ddd	9.9, 5.1, 2.3	1$_{C}$
	**6**	60.72	T	3.90^{H}	1	m	-	
				3.78^{H}	1	m	-	
	**2-CO**	175.16	S	-	0	-	-	2^{C}, Ac
	**Ac**	22.43	Q	2.042	3	s	-	
**Gal$_{D}$**	**1**	103.67	D	4.442	1	d	7.8	
	**2**	70.89	D	3.527	1	dd	10.0, 7.8	
	**3**	72.68	D	3.642	1	dd	10.0, 3.4	
	**4**	68.73	D	3.915	1	br d	3.4	
	**5**	75.48	D	3.70^{H}	1	m	-	1$_{D}$

	**6**	61.21	T	3.76[H]	2	m	-	

β-anomer

	**Atom**	C	**m.**	H	**n**H	**m.**	***J*** **[Hz]**	**HMBC**[x]
**Glc^{A}**	**1**	95.94	D	4.662	1	d	7.9	
	**2**	74.00	D	3.281	1	m	-	
	**3**	74.56	D	3.664	1	m	-	
	**4**	78.62	D	3.664	1	m	-	1^{B}
	**5**	74.99	D	3.600	1	m	-	1^{A}
	**6**	60.31	T	3.95[H]	1	m	-	
				3.79[H]	1	m	-	
**Gal^{B}**	**1**	103.12	D	4.443	1	d	7.9	
	**2**	70.21	D	3.594	1	dd	10.0, 7.9	
	**3**	82.18	D	3.734	1	dd	10.0, 3.2	1^{C}
	**4**	68.51	D	4.151	1	br d	3.3	
	**5**	75.09	D	3.71[H]	1	m	-	1^{B}
	**6**	61.14	T	3.78[H]	1	m	-	
				3.74[H]	1	m	-	
**GlcNAc^{C}**	**1**	102.72	D	4.738	1	d	8.4	
	**2**	54.90	D	3.896	1	dd	10.3, 8.4	
	**3**	82.31	D	3.820	1	dd	10.3, 8.5	1^{D}
	**4**	68.68	D	3.571	1	dd	9.9, 8.5	
	**5**	75.40	D	3.483	1	ddd	9.9, 5.1, 2.3	1^{C}

	**6**	60.72	T	3.90^H	1	m	-	
				3.78^H	1	m	-	
	**2-CO**	175.16	S	-	0	-	-	2^C, Ac
	**Ac**	22.43	Q	2.042	3	s	-	
**Gal$_D$**	**1**	103.67	D	4.442	1	d	7.8	
	**2**	70.89	D	3.527	1	dd	10.0, 7.8	
	**3**	72.68	D	3.642	1	dd	10.0, 3.4	
	**4**	68.73	D	3.915	1	br d	3.4	
	**5**	75.48	D	3.70^H	1	m	-	1^D
	**6**	61.21	T	3.76^H	2	m	-	

^{H} HSQC readout; ^{x} diagnostic C to H HMBC correlations

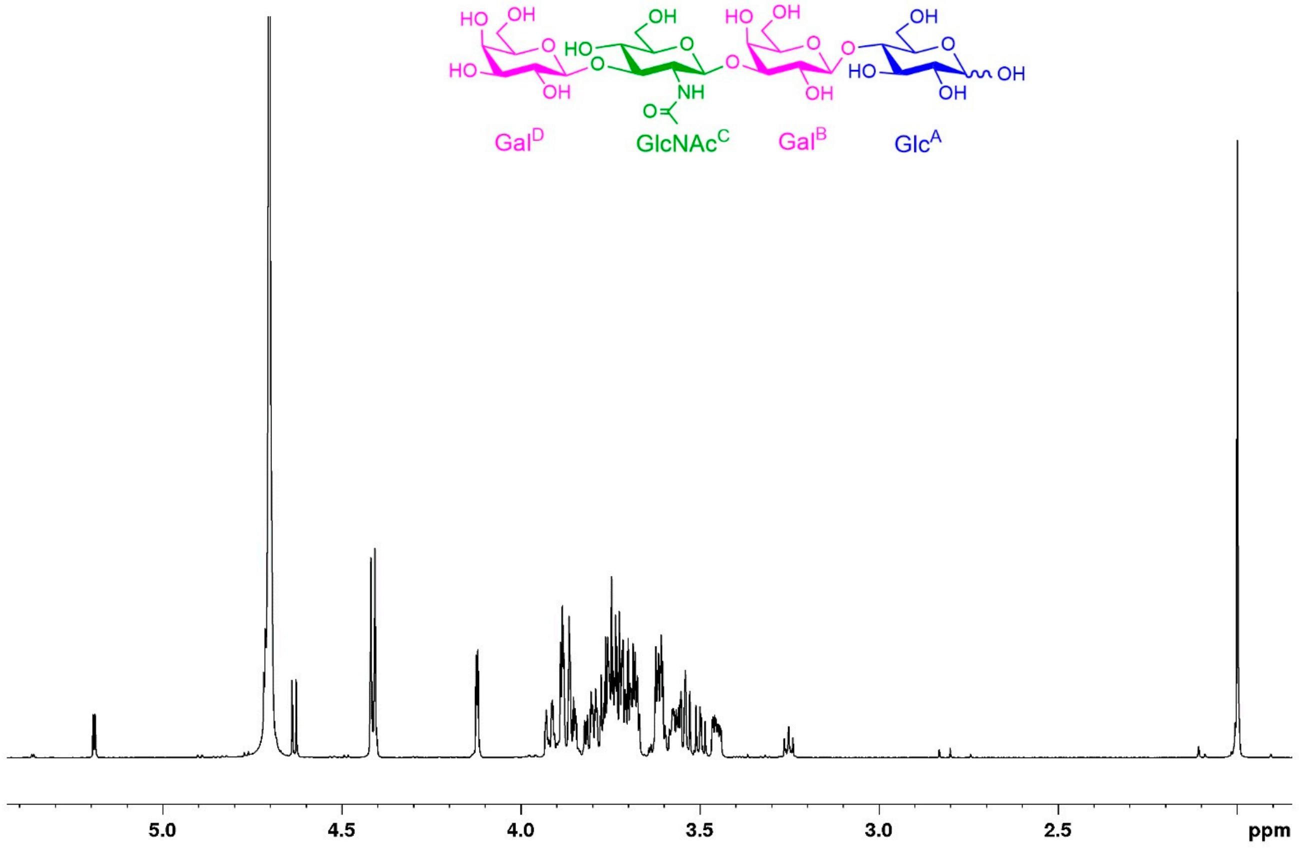

Figure S3a. ^{1}H NMR spectrum of compound **5** (700.13 MHz for ^{1}H, D_2O, 30 °C).

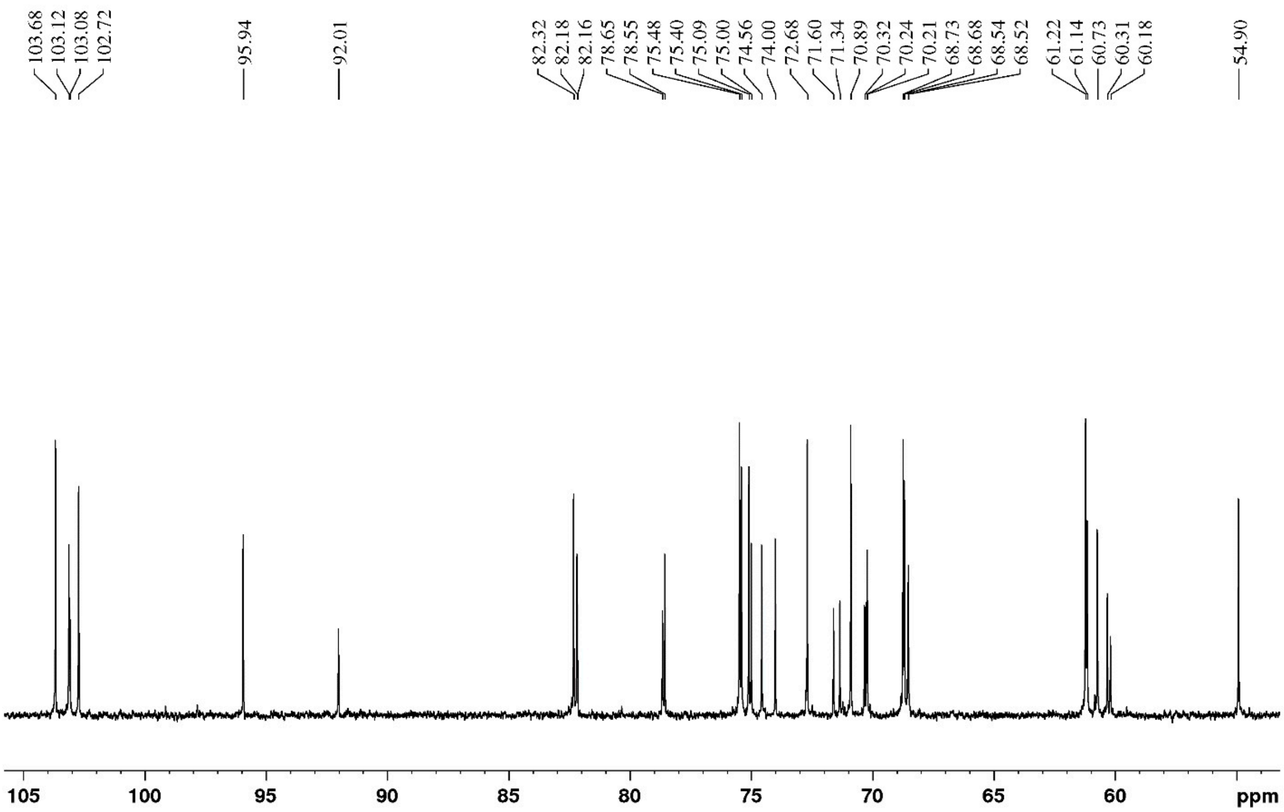

Figure S3b. ^{13}C NMR spectrum of compound **5** – without signals of *N*-acetyls (176.05 MHz for ^{13}C, D_2O, 30 °C).

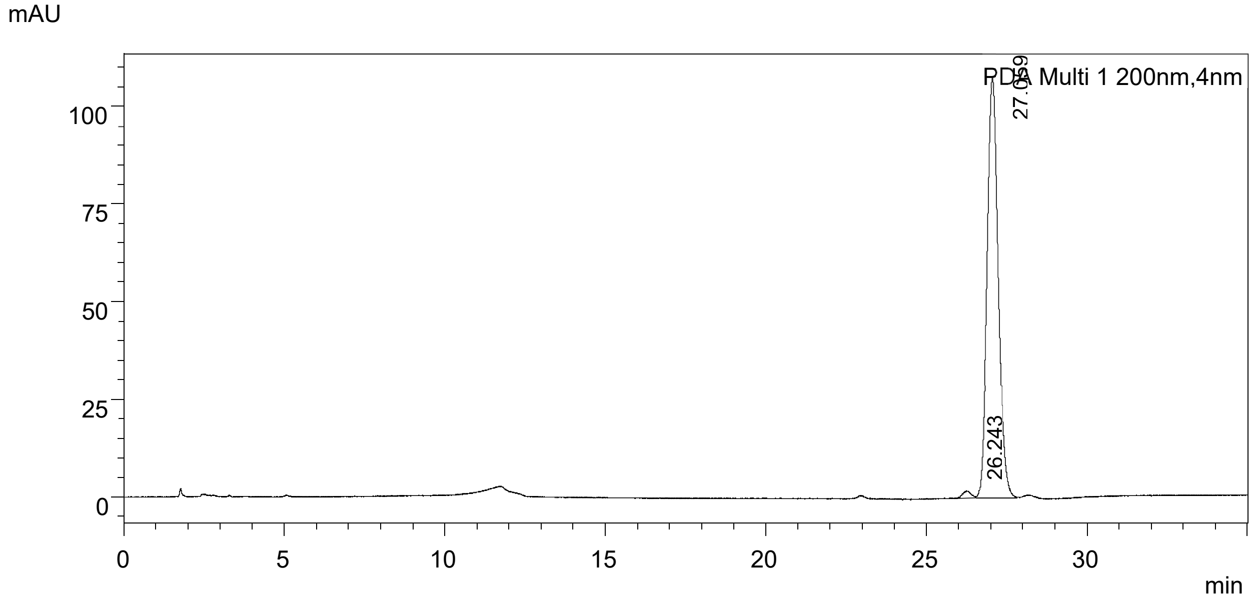

Figure S3c. HPLC chromatogram of isolated compound **5** (RT= 27.059, 98% purity).

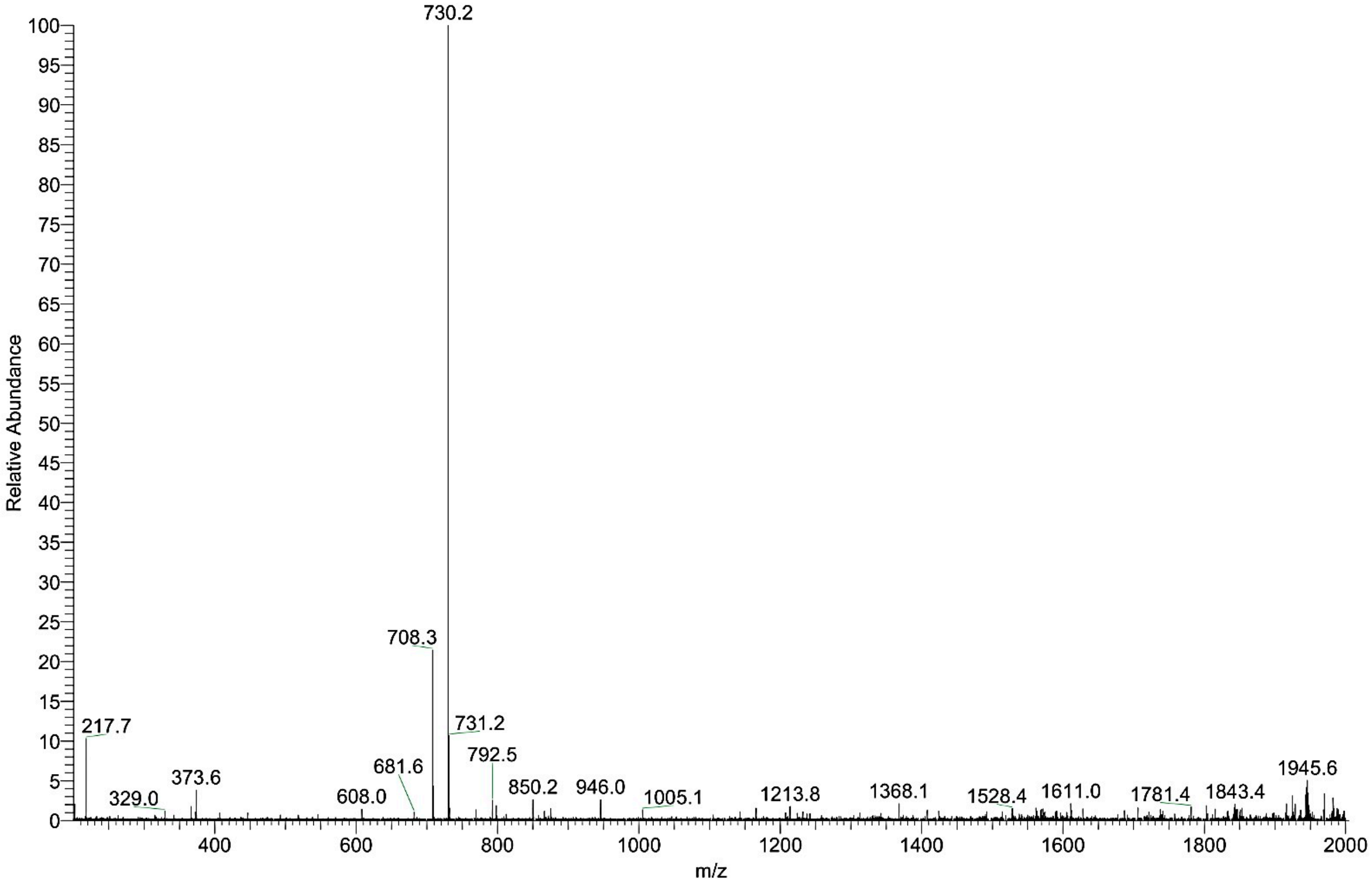

Figure S3d. MS spectrum (ESI+) of compound **5**: [M + H]+, *m/z* 708.3; [M + Na]+, *m/z* 730.2. HRMS spectrum (ESI+): *m/z* for $C_{26}H_{45}O_{21}NNa^+$ calculated 730.23763, found 730.23752 (-0.14 ppm).

Table S3. ^{1}H and ^{13}C NMR data for β-D-galactopyranosyl-(1→4)-2-acetamido-2-deoxy-β-D-glucopyranosyl-(1→3)-β-Dgalactopyranosyl-(1→4)-D-glucopyranose (**7**; 700.13 MHz for ^{1}H, 176.05 MHz for ^{13}C, D_2O, 30 °C). ***α*-anomer**

	**Atom**	C	**m.**	H	n_H	**m.**	***J* [Hz]**	**HMBC^x**
**Glc^A**	**1**	92.08	D	5.226	1	d	3.8	
	**2**	71.41	D	3.582	1	dd	9.9, 3.8	
	**3**	71.67	D	3.836	1	dd	9.9, 8.9	
	**4**	78.71	D	3.645	1	dd	10.0, 8.9	1^B
	**5**	70.39	D	3.953	1	ddd	-	1^A
	**6**	60.25	T	3.89^H	1	m	-	
				3.85^H	1	m	-	

**Gal[B]**	**1**	103.16	D	4.447	1	d	7.9	
	**2**	70.27	D	3.600	1	dd	9.9, 7.9	
	**3**	82.28	D	3.727	1	dd	9.9, 3.2	1[C]
	**4**	68.63	D	4.158	1	br d	3.4	
	**5**	75.15	D	3.73[H]	1	m	-	1[B]
	**6**	61.21	T	3.78[H]	1	m	-	
				3.74[H]	1	m	-	
**GlcNAc[C]**	**1**	102.97	D	4.720	1	d	8.3	
	**2**	55.48	D	3.808	1	dd	10.4, 8.3	
	**3**	72.46	D	3.74[H]	1	m	-	
	**4**	78.52	D	3.74[H]	1	m	-	1[D]
	**5**	74.83	D	3.59[H]	1	m	-	1[C]
	**6**	60.18	T	3.96[H]	1	m	-	
				3.85[H]	1	m	-	
	**2-CO**	175.17	S	-	0	-	-	2[C], Ac
	**Ac**	22.45	Q	2.040	3	s	-	
**Gal[D]**	**1**	103.14	D	4.485	1	d	7.8	
	**2**	71.24	D	3.548	1	dd	10.0, 7.8	
	**3**	73.00	D	3.674	1	dd	10.0, 3.4	
	**4**	68.82	D	3.934	1	br d	3.4	
	**5**	75.62	D	3.73[H]	1	m	-	1[D]
	**6**	61.28	T	3.77[H]	2	m	-	

β-anomer

	Atom	C	m.	H	n_H	m.	*J* [Hz]	HMBC^x
**Glc^A**	**1**	96.01	D	4.668	1	d	7.9	
	**2**	74.07	D	3.287	1	m	-	
	**3**	74.63	D	3.650	1	m	-	
	**4**	78.61	D	3.650	1	m	-	1^B
	**5**	75.07	D	3.606	1	m	-	1^A
	**6**	60.38	T	3.956	1	m	12.3, 2.3	
				3.798	1	dd	12.3, 5.2	
**Gal^B**	**1**	103.20	D	4.445	1	d	7.9	
	**2**	70.24	D	3.597	1	dd	9.9, 7.9	
	**3**	82.30	D	3.727	1	dd	9.9, 3.2	1^C
	**4**	68.60	D	4.158	1	br d	3.4	
	**5**	75.15	D	3.73^H	1	m	-	1^B
	**6**	61.21	T	3.78^H	1	m	-	
				3.74^H	1	m	-	
**GlcNAc^C**	**1**	102.97	D	4.716	1	d	8.3	
	**2**	55.48	D	3.808	1	dd	10.4, 8.3	
	**3**	72.46	D	3.74^H	1	m	-	
	**4**	78.52	D	3.74^H	1	m	-	1^D
	**5**	74.83	D	3.59^H	1	m	-	1^C
	**6**	60.18	T	3.96^H	1	m	-	
				3.85^H	1	m	-	

	**2-CO**	175.17	S	-	0	-	-	2^{C}, Ac
	**Ac**	22.45	Q	2.040	3	s	-	
**Gal$_{D}$**	**1**	103.14	D	4.485	1	d	7.8	
	**2**	71.24	D	3.548	1	dd	10.0, 7.8	
	**3**	73.00	D	3.674	1	dd	10.0, 3.4	
	**4**	68.82	D	3.934	1	br d	3.4	
	**5**	75.62	D	3.73^{H}	1	m	-	1^{D}
	**6**	61.28	T	3.77^{H}	2	m	-	

^{H} HSQC readout; ^{x} diagnostic C to H HMBC correlations

Table S4. ^{1}H and ^{13}C NMR data for β-D-galactopyranosyl-(1→6)-2-acetamido-2-deoxy-β-D-glucopyranosyl-(1→3)-β-Dgalactopyranosyl-(1→4)-D-glucopyranose (**7a**; 700.13 MHz for ^{1}H, 176.05 MHz for ^{13}C, D_2O, 30 °C).

***α*-anomer**

	**Atom**	C	**m.**	H	**n$_{H}$**	**m.**	***J* [Hz]**	**HMBC^{x}**
**Glc^{A}**	**1**	92.08	D	5.226	1	d	3.8	
	**2**	71.41	D	3.582	1	dd	9.9, 3.8	
	**3**	71.67	D	3.836	1	dd	9.9, 8.9	
	**4**	78.71	D	3.645	1	dd	10.0, 8.9	1^{B}
	**5**	70.39	D	3.953	1	ddd	-	1^{A}
	**6**	60.25	T	3.89^{H}	1	m	-	
				3.85^{H}	1	m	-	
**Gal^{B}**	**1**	103.16	D	4.447	1	d	7.9	
	**2**	70.34	D	3.60^{H}	1	m	-	
	**3**	82.21	D	3.73^{H}	1	m	-	1^{C}

	**4**	68.57	D	4.171	1	br d	3.4	
	**5**	75.15	D	3.72^{H}	1	m	-	1^{B}
	**6**	61.23	T	3.78^{H}	1	m	-	
				3.74^{H}	1	m	-	
**GlcNAc^{C}**	**1**	103.11	D	4.704	1	d	8.4	
	**2**	55.91	D	3.767	1	dd	-	
	**3**	73.78	D	3.583	1	dd	-	
	**4**	69.87	D	3.557	1	dd	-	
	**5**	74.95	D	3.619	1	ddd	9.8, 5.5, 1.9	1^{C}
	**6**	68.81	T	4.223	1	dd	11.7, 1.9	1^{D}
				3.881	1	dd	11.7, 5.5	
	**2-CO**	175.22	S	-	0	-	-	2^{C}, Ac
	**Ac**	22.44	Q	2.044	3	s	-	
**Gal$_{D}$**	**1**	103.82	D	4.443	1	d	7.9	6^{C}
	**2**	71.03	D	3.556	1	dd	10.0, 7.9	
	**3**	73.00	D	3.65	1	m	-	
	**4**	68.90	D	3.93	1	m	-	
	**5**	75.42	D	3.698	1	ddd	7.8, 4.5, 1.0	1^{D}
	**6**	61.26	T	3.77^{H}	2	m	-	

β-anomer

	**Atom**	$_{C}$	**m.**	$_{H}$	**n_H**	**m.**	***J* [Hz]**	**HMBC^{x}**
**Glc^{A}**	**1**	96.01	D	4.668	1	d	7.9	
	**2**	74.07	D	3.287	1	m	-	

	**3**	74.63	D	3.650	1	m	-	
	**4**	78.61	D	3.650	1	m	-	1[B]
	**5**	75.07	D	3.606	1	m	-	1[A]
	**6**	60.38	T	3.956	1	m	12.3, 2.3	
				3.798	1	dd	12.3, 5.2	
**Gal[B]**	**1**	103.20	D	4.445	1	d	7.9	
	**2**	70.31	D	3.60[H]	1	m	-	
	**3**	82.24	D	3.73[H]	1	m	-	1[C]
	**4**	68.57	D	4.171	1	br d	3.4	
	**5**	75.15	D	3.72[H]	1	m	-	1[B]
	**6**	61.23	T	3.78[H]	1	m	-	
				3.74[H]	1	m	-	
**GlcNAc[C]**	**1**	103.11	D	4.704	1	d	8.4	
	**2**	55.91	D	3.767	1	dd	-	
	**3**	73.78	D	3.583	1	dd	-	
	**4**	69.87	D	3.557	1	dd	-	
	**5**	74.95	D	3.619	1	ddd	9.8, 5.5, 1.9	1[C]
	**6**	68.81	T	4.223	1	dd	11.7, 1.9	1[D]
				3.881	1	dd	11.7, 5.5	
	**2-CO**	175.22	S	-	0	-	-	2[C], Ac
	**Ac**	22.44	Q	2.044	3	s	-	
**Gal$_D$**	**1**	103.82	D	4.443	1	d	7.9	6[C]

	**2**	71.03	D	3.556	1	dd	10.0, 7.9	
	**3**	73.00	D	3.65	1	m	-	
	**4**	68.90	D	3.93	1	m	-	
	**5**	75.42	D	3.698	1	ddd	7.8, 4.5, 1.0	1[D]
	**6**	61.26	T	3.77[H]	2	m	-	

H HSQC readout; x diagnostic ^{13}C to ^{1}H HMBC correlations D

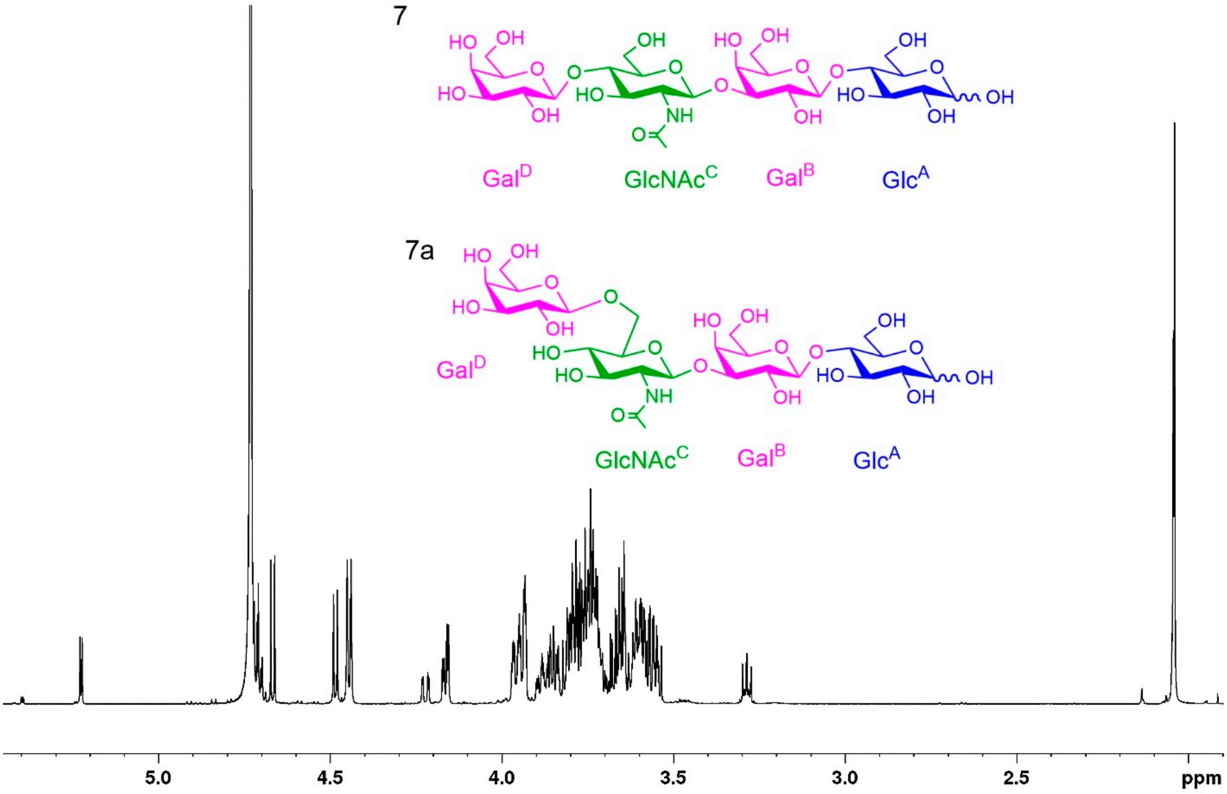

Figure S4a. ^{1}H NMR spectrum of compound **7** containing 35% of β(1→6)-linked side product **7a** (700.13 MHz for 1H,

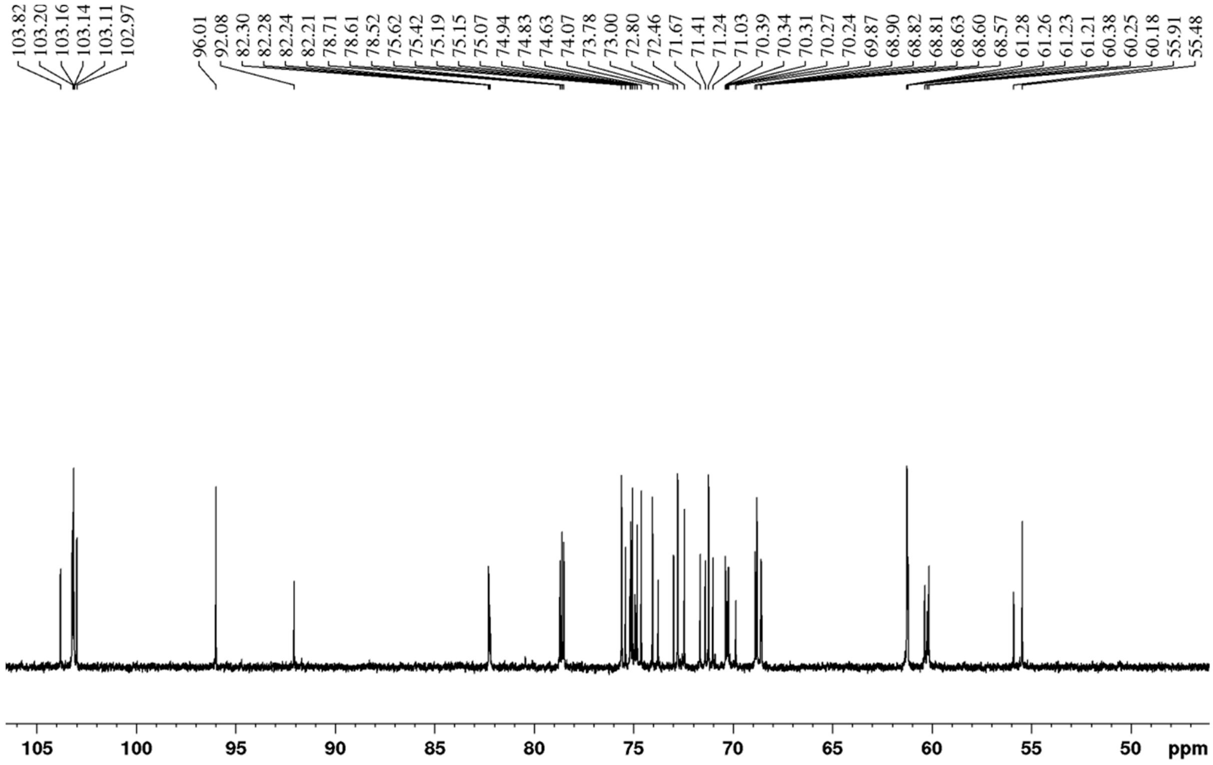

2O, 30 °C).

Figure S4b. ^{13}C NMR spectrum of compound **7** containing 35% of β(1→6)-linked side product **7a** – without signals of *N*-acetyls (176.05 MHz for ^{13}C, D_2O, 30 °C).

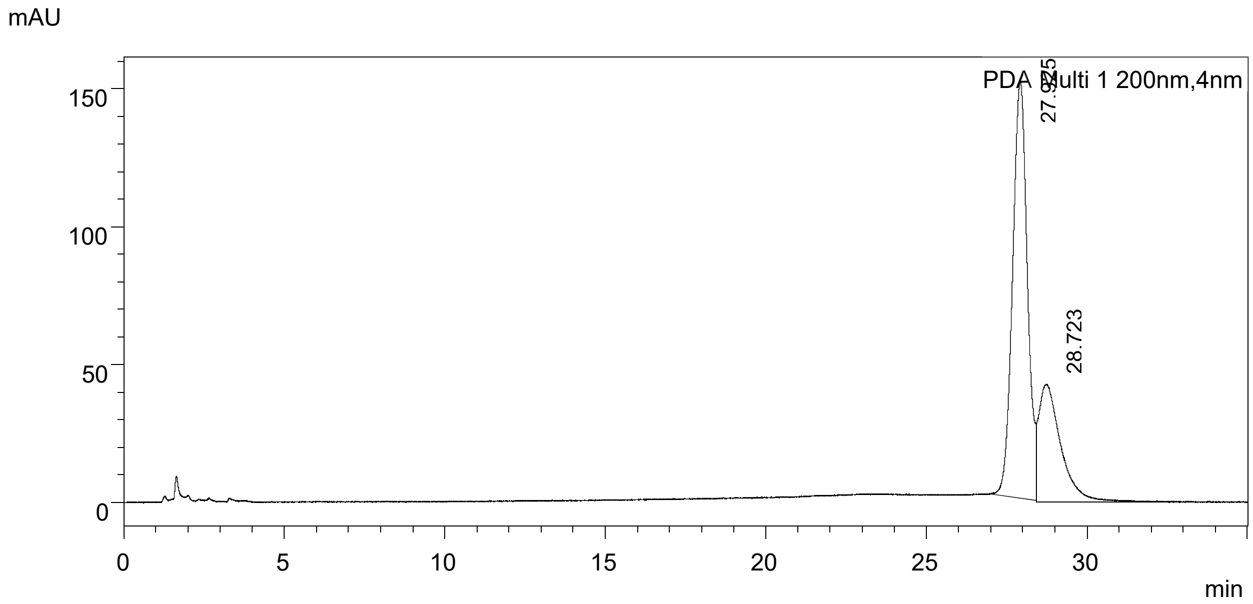

Figure S4c. HPLC chromatogram of compound **7** containing 35% of β(1→6)-linked side product **7a** (RT= 27.925 and 28.823 min for α- and β-anomers, 99% purity). Compounds **7** and **7a** do not separate on HPLC.

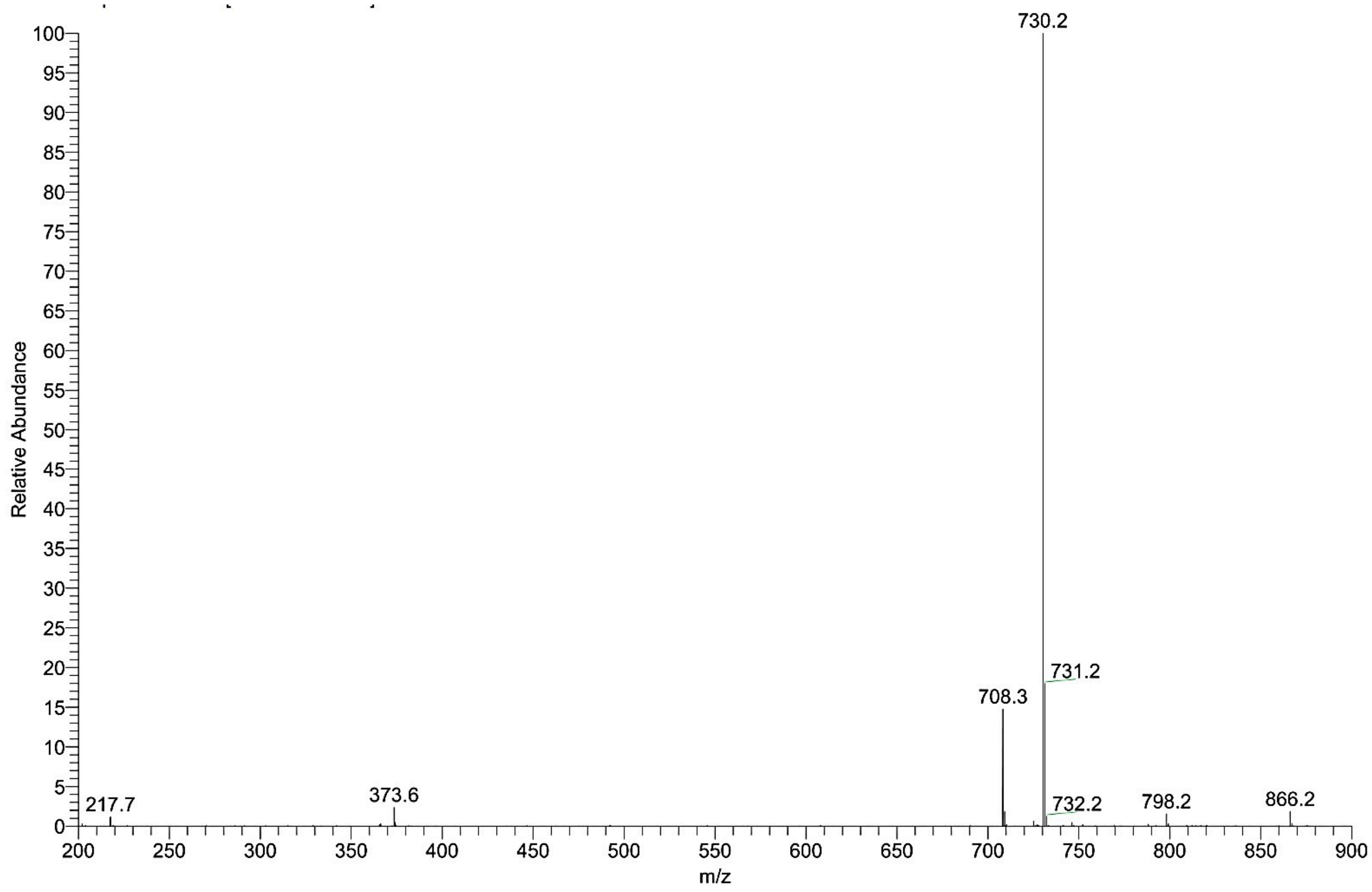

Figure S4d. MS spectrum (ESI+) of compound **7** containing 35% of β(1→6)-linked side product **7a**: $[M + H]^+$, *m/z* 708.3; $[M + Na]^+$, *m/z* 730.2. HRMS spectrum (ESI+): *m/z* for $C_{26}H_{45}O_{21}NNa^+$ calculated 730.23763, found 730.23743 (-0.27 ppm).

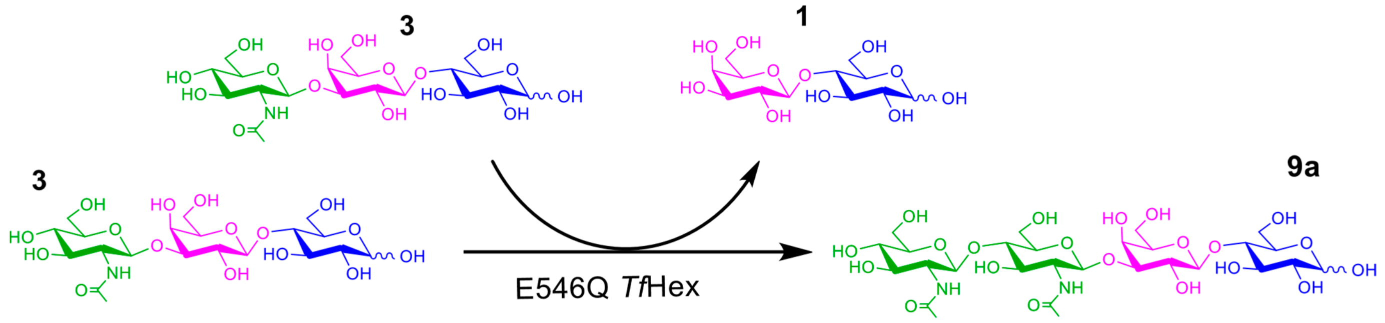

Scheme S1. Side reaction in the synthesis of product **9** catalyzed by E546Q *Tf*Hex, leading to GlcNAc-capped sideproduct **9a**.

Table S5. ^{1}H and ^{13}C NMR data for 2-acetamido-2-deoxy-β-D-galactopyranosyl-(1→4)-2-acetamido-2-deoxy-β-Dglucopyranosyl-(1→3)-β-D-galactopyranosyl-(1→4)-D-glucopyranose (**9**; 700.13 MHz for ^{1}H, 176.05 MHz for ^{13}C, D_2O, 30 °C).

***α*-anomer**

	**Atom**	C	**m.**	H	**n_H**	**m.**	***J* [Hz]**	**HMBC^{x}**
**Glc^{A}**	**1**	92.09	D	5.226	1	d	3.8	
	**2**	71.42	D	3.581	1	dd	9.9, 3.8	
	**3**	71.67	D	3.834	1	dd	9.9, 8.8	
	**4**	78.71	D	3.644	1	dd	10.1, 8.8	1^{B}
	**5**	70.39	D	3.952	1	ddd	-	1^{A}
	**6**	60.25	T	3.89^{H}	1	m	-	
				3.84^{H}	1	m	-	
**Gal^{B}**	**1**	103.16	D	4.444	1	d	7.9	
	**2**	70.26	D	3.597	1	dd	10.0, 7.9	
	**3**	82.30	D	3.72^{H}	1	m	-	1^{C}
	**4**	68.61	D	4.143	1	br d	3.3	
	**5**	75.12	D	3.72^{H}	1	m	-	1^{B}
	**6**	61.20	T	3.78^{H}	1	m	-	
				3.74^{H}	1	m	-	
**GlcNAc^{C}**	**1**	102.95	D	4.689	1	d	8.3	

	**2**	55.23	D	3.796	1	dd	10.4, 8.3	
	**3**	72.56	D	3.743	1	dd	-	
	**4**	79.22	D	3.653	1	dd	-	1^{D}
	**5**	74.59	D	3.519	1	ddd	9.9, 4.9, 2.1	1^{C}
	**6**	60.25	T	3.84^{H}	1	m	-	
				3.66^{H}	1	m	-	
	**2-CO**	175.18	S	-	0	-	-	2^{C}, Ac
	**Ac**	22.44	Q	2.036	3	s	-	
**GalNAc^{D}**	**1**	101.99	D	4.533	1	d	8.4	
	**2**	52.82	D	3.936	1	dd	10.7, 8.4	
	**3**	70.94	D	3.758	1	dd	10.7, 3.3	
	**4**	67.87	D	3.950	1	m	-	
	**5**	75.58	D	3.74^{H}	1	m	-	1^{D}
	**6**	61.20	T	3.78^{H}	1	m	-	
				3.74^{H}	1	m	-	
	**2-CO**	175.04	S	-	0	-	-	2^{D}, Ac
	**Ac**	22.46	T	2.073	3	s	-	

β-anomer

	**Atom**	C	**m.**	H	**n$_{H}$**	**m.**	***J* [Hz]**	**HMBC^{x}**
**Glc^{A}**	**1**	96.04	D	4.667	1	d	7.9	
	**2**	74.09	D	3.285	1	m	-	
	**3**	74.63	D	3.648	1	m	-	
	**4**	78.61	D	3.648	1	m	-	1^{B}
	**5**	75.06	D	3.601	1	m	-	1^{A}
	**6**	60.37	T	3.95^{H}	1	m	-	

				3.80[H]	1	m	-	
**Gal[B]**	**1**	103.19	D	4.442	1	d	7.9	
	**2**	70.24	D	3.589	1	dd	10.0, 7.9	
	**3**	82.31	D	3.72[H]	1	m	-	1[C]
	**4**	68.58	D	4.143	1	br d	3.3	
	**5**	75.12	D	3.72[H]	1	m	-	1[B]
	**6**	61.20	T	3.78[H]	1	m	-	
				3.74[H]	1	m	-	
**GlcNAc[C]**	**1**	102.95	D	4.693	1	d	8.3	
	**2**	55.23	D	3.796	1	dd	10.4, 8.3	
	**3**	72.56	D	3.743	1	dd	-	
	**4**	79.22	D	3.653	1	dd	-	1[D]
	**5**	74.59	D	3.519	1	ddd	9.9, 4.9, 2.1	1[C]
	**6**	60.25	T	3.84[H]	1	m	-	
				3.66[H]	1	m	-	
	**2-CO**	175.18	S	-	0	-	-	2[C], Ac
	**Ac**	22.44	Q	2.036	3	s	-	
**GalNAc[D]**	**1**	101.99	D	4.533	1	d	8.4	
	**2**	52.82	D	3.936	1	dd	10.7, 8.4	
	**3**	70.94	D	3.758	1	dd	10.7, 3.3	
	**4**	67.87	D	3.950	1	m	-	
	**5**	75.58	D	3.74[H]	1	m	-	1[D]

	**6**	61.20	T	3.78^{H}	1	m	-	
				3.74^{H}	1	m	-	
	**2-CO**	175.04	S	-	0	-	-	2^{D}, Ac
	**Ac**	22.46	T	2.073	3	s	-	

^{H} HSQC readout; ^{x} diagnostic C to H HMBC correlations **Table S6.** ^{1}H and ^{13}C NMR data for 2-acetamido-2-deoxy-β-D-glucopyranosyl-(1→4)-2-acetamido-2-deoxy-β-Dglucopyranosyl-(1→3)-β-D-galactopyranosyl-(1→4)-D-glucopyranose (**9a**; 700.13 MHz for ^{1}H, 176.05 MHz for ^{13}C, D_2O, 30 °C).

α-anomer

	**Atom**	C	**m.**	H	n_H	**m.**	***J* [Hz]**	**HMBC^{x}**
**Glc^{A}**	**1**	92.09	D	5.226	1	d	3.8	
	**2**	71.42	D	3.581	1	dd	9.9, 3.8	
	**3**	71.67	D	3.834	1	dd	9.9, 8.8	
	**4**	78.71	D	3.644	1	dd	10.1, 8.8	1^{B}
	**5**	70.39	D	3.952	1	ddd	-	1^{A}
	**6**	60.25	T	3.89^{H}	1	m	-	
				3.84^{H}	1	m	-	
**Gal^{B}**	**1**	103.16	D	4.444	1	d	7.9	
	**2**	70.26	D	3.597	1	dd	10.0, 7.9	
	**3**	82.30	D	3.72^{H}	1	m	-	1^{C}
	**4**	68.61	D	4.143	1	br d	3.3	
	**5**	75.12	D	3.72^{H}	1	m	-	1^{B}
	**6**	61.20	T	3.78^{H}	1	m	-	
				3.74^{H}	1	m	-	
**GlcNAc^{C}**	**1**	102.95	D	4.689	1	d	8.3	

	**2**	55.33	D	3.790	1	dd	10.4, 8.4	
	**3**	72.50	D	3.727	1	dd	-	
	**4**	79.53	D	3.640	1	dd	-	1[D]
	**5**	74.57	D	3.511	1	ddd	9.9, 4.9, 2.1	1[C]
	**6**	60.25	T	3.84[H]	1	m	-	
				3.66[H]	1	m	-	
	**2-CO**	175.18	S	-	0	-	-	2[C], Ac
	**Ac**	22.44	Q	2.036	3	s	-	
**GlcNAc[D]**	**1**	101.74	D	4.597	1	d	8.5	
	**2**	55.85	D	3.755	1	dd	10.4, 8.5	
	**3**	73.72	D	3.576	1	dd	10.4, 8.7	
	**4**	69.98	D	3.477	1	dd	9.9, 8.7	
	**5**	76.15	D	3.512	1	ddd	9.9, 5.6, 2.2	1[D]
	**6**	60.80	T	3.93[H]	1	m	-	
				3.75[H]	1	m	-	
	**2-CO**	174.88	S	-	0	-	-	2[D], Ac
	**Ac**	22.39	T	2.073	3	s	-	

β-anomer

	**Atom**	C	**m.**	H	**n_H**	**m.**	***J* [Hz]**	**HMBC[x]**
**Glc[A]**	**1**	96.04	D	4.667	1	d	7.9	
	**2**	74.09	D	3.285	1	m	-	
	**3**	74.63	D	3.648	1	m	-	

	**4**	78.61	D	3.648	1	m	-	1^{B}
	**5**	75.06	D	3.601	1	m	-	1^{A}
	**6**	60.37	T	3.95^{H}	1	m	-	
				3.80^{H}	1	m	-	
**Gal^{B}**	**1**	103.19	D	4.442	1	d	7.9	
	**2**	70.24	D	3.589	1	dd	10.0, 7.9	
	**3**	82.31	D	3.72^{H}	1	m	-	1^{C}
	**4**	68.58	D	4.143	1	br d	3.3	
	**5**	75.12	D	3.72^{H}	1	m	-	1^{B}
	**6**	61.20	T	3.78^{H}	1	m	-	
				3.74^{H}	1	m	-	
**GlcNAc^{C}**	**1**	102.95	D	4.693	1	d	8.3	
	**2**	55.33	D	3.790	1	dd	10.4, 8.4	
	**3**	72.50	D	3.727	1	dd	-	
	**4**	79.53	D	3.640	1	dd	-	1^{D}
	**5**	74.57	D	3.511	1	ddd	9.9, 4.9, 2.1	1^{C}
	**6**	60.25	T	3.84^{H}	1	m	-	
				3.66^{H}	1	m	-	
	**2-CO**	175.18	S	-	0	-	-	2^{C}, Ac
	**Ac**	22.44	Q	2.036	3	s	-	
**GlcNAc^{D}**	**1**	101.74	D	4.597	1	d	8.5	
	**2**	55.85	D	3.755	1	dd	10.4, 8.5	

	**3**	73.72	D	3.576	1	dd	10.4, 8.7	
	**4**	69.98	D	3.477	1	dd	9.9, 8.7	
	**5**	76.15	D	3.512	1	ddd	9.9, 5.6, 2.2	1[D]
	**6**	60.80	T	3.93[H]	1	m	-	
				3.75[H]	1	m	-	
	**2-CO**	174.88	S	-	0	-	-	2[D], Ac
	**Ac**	22.39	T	2.073	3	s	-	

[H] HSQC readout; [x] diagnostic ^{13}C to ^{1}H HMBC correlations

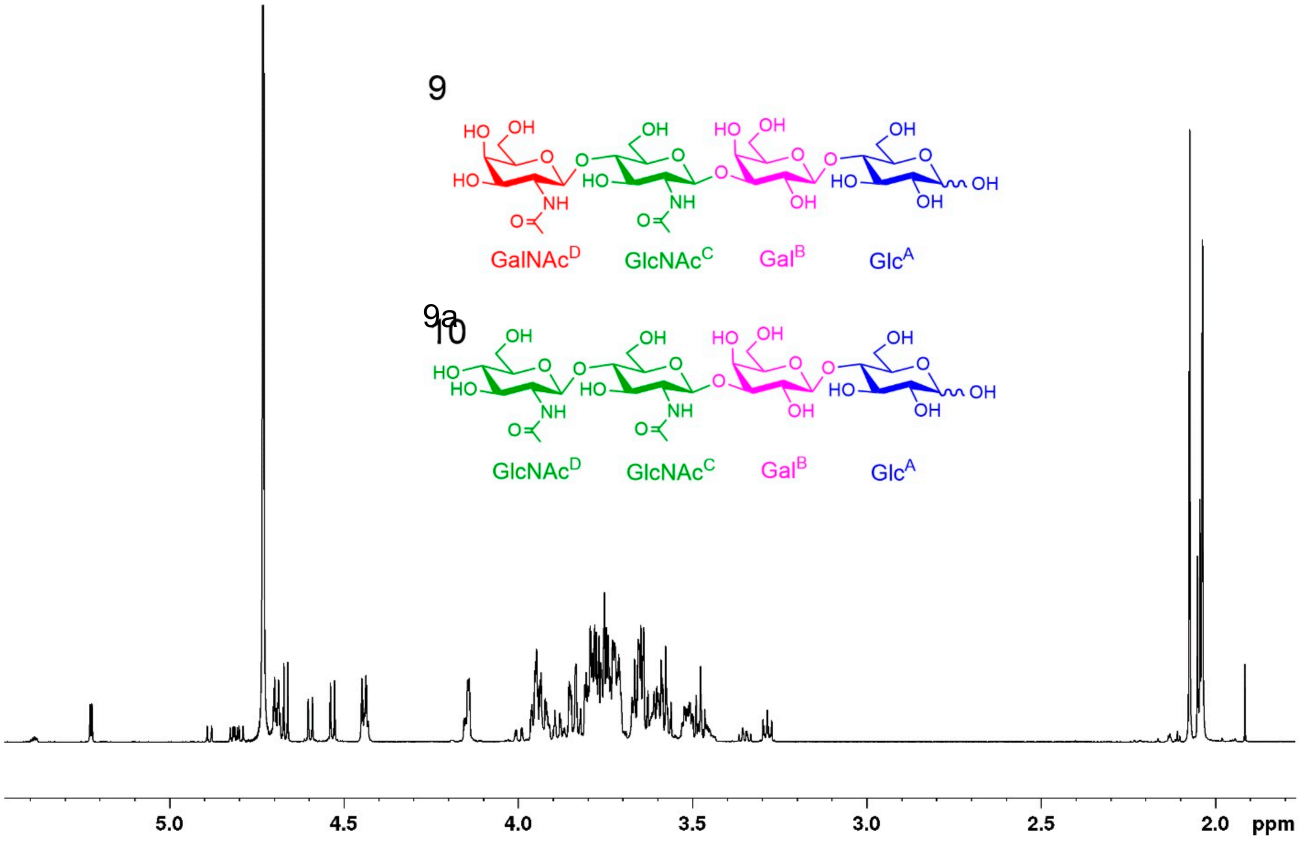

Figure S5a. ^{1}H NMR spectrum of compounds **9** containing 44% of GlcNAc-capped side product **9a** (700.13 MHz for ^{1}H, D_2O, 30 °C).

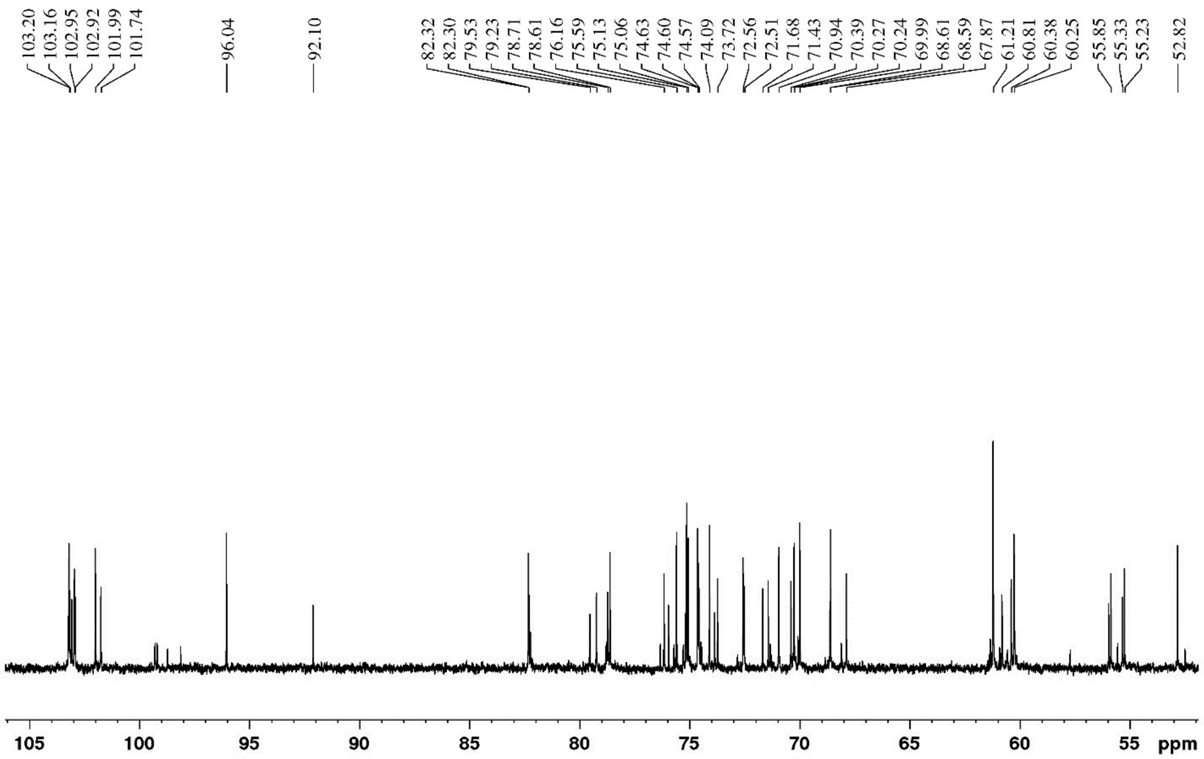

Figure S5b. ^{13}C NMR spectrum of compounds **9** containing 44% of GlcNAc-capped side product **9a** – without signals of *N*-acetyls (176.05 MHz for ^{13}C, D_2O, 30 °C).

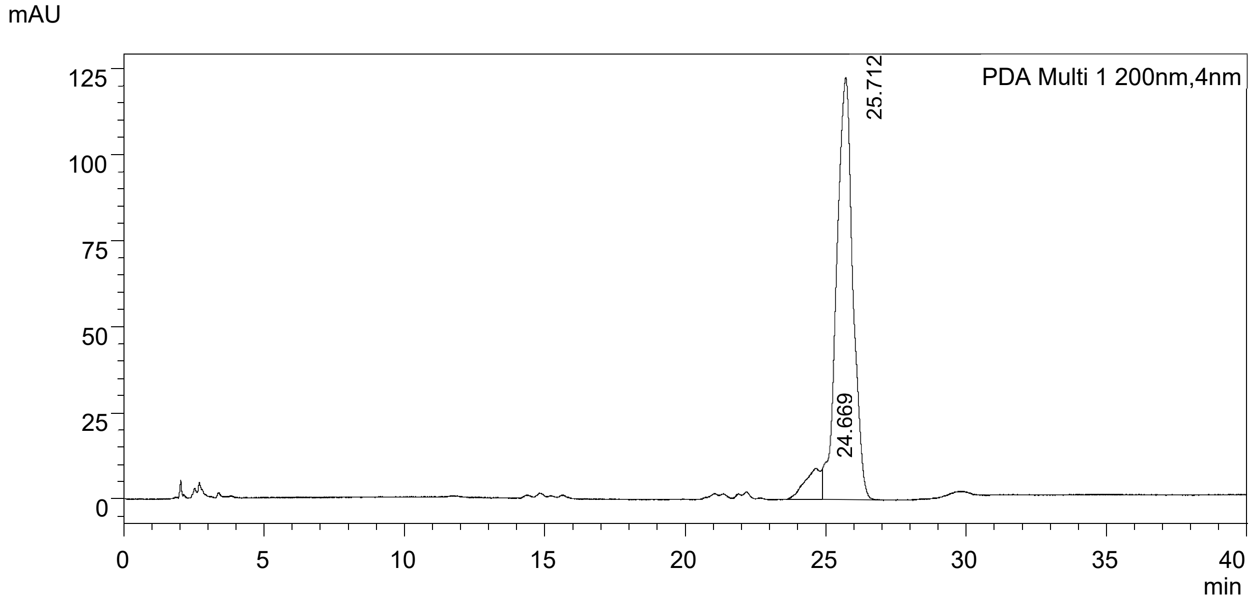

Figure S5c. HPLC chromatogram of isolated compound **9** containing 44% of GlcNAc-capped side product **9a** (RT= 24.669 and 25.712 min for α- and β-anomers, 99% purity). Compounds **9** and **9a** do not separate on HPLC.

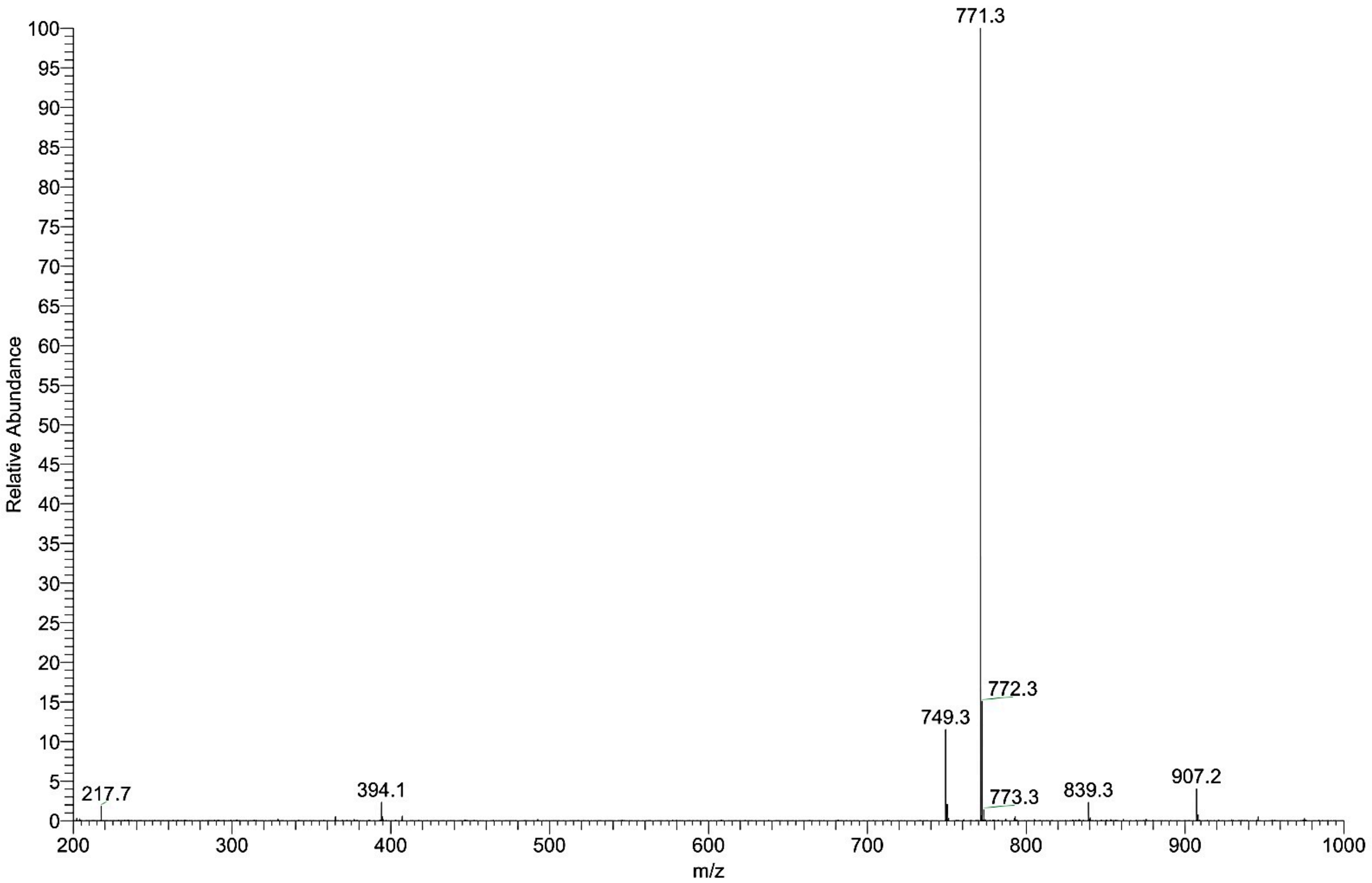

Figure S5d. MS spectrum (ESI+) of compound **9** containing 44% of GlcNAc-capped side product **9a**: [M + H]+, *m/z* 749.3; [M + Na]+, *m/z* 771.3. MS spectrum (ESI+): *m/z* for $C_{28}H_{48}O_{21}N_2Na^+$ calculated 771.26418, found 771.26407 (-0.14 ppm).

4. References

1. Hovorková, M.; Kulik, N.; Konvalinková, D.; Petrásková, L.; Křen, V.; Bojarová, P., Mutagenesis of Catalytic Nucleophile of β-Galactosidase Retains Residual Hydrolytic Activity and Affords a Transgalactosidase. *ChemCatChem* **2021,** 13, (21), 4532-4542.
